# Short Chain Fatty Acids Prevent Glyoxylate-Induced Calcium Oxalate Stones by GPR43-Dependent Immunomodulatory Mechanism

**DOI:** 10.3389/fimmu.2021.729382

**Published:** 2021-10-05

**Authors:** Xi Jin, Zhongyu Jian, Xiaoting Chen, Yucheng Ma, Hongwen Ma, Yu Liu, Lina Gong, Liyuan Xiang, Shiyu Zhu, Xiaoling Shu, Shiqian Qi, Hong Li, Kunjie Wang

**Affiliations:** ^1^ Department of Urology, Institute of Urology (Laboratory of Reconstructive Urology), West China Hospital, Sichuan University, Chengdu, China; ^2^ Animal Experimental Center, West China Hospital, Sichuan University, Chengdu, China; ^3^ State Key Laboratory of Biotherapy and Cancer Center, West China Hospital, Sichuan University, Chengdu, China

**Keywords:** calcium oxalate stones, short chain fatty acid, macrophage, neutrophil, GPR43

## Abstract

Calcium oxalate (CaOx) stones are the most common type of kidney stones and are associated with high recurrence, short chain fatty acids (SCFAs), and inflammation. However, it remains uncertain whether SCFAs affect the formation of CaOx stones through immunomodulation. We first performed mass cytometry (CyTOF) and RNA sequencing on kidney immune cells with glyoxylate-induced CaOx crystals (to elucidate the landscape of the associated immune cell population) and explored the role of SCFAs in renal CaOx stone formation through immunomodulation. We identified 29 distinct immune cell subtypes in kidneys with CaOx crystals, where CX3CR1^+^CD24^-^ macrophages significantly decreased and GR1^+^ neutrophils significantly increased. In accordance with the CyTOF data, RNA sequencing showed that most genes involved were related to monocytes and neutrophils. SCFAs reduced kidney CaOx crystals by increasing the frequency of CX3CR1^+^CD24^-^ macrophages and decreasing GR1^+^ neutrophil infiltration in kidneys with CaOx crystals, which was dependent on the gut microbiota. GPR43 knockdown by transduction with adeno-associated virus inhibited the alleviation of crystal formation and immunomodulatory effects in the kidney, due to SCFAs. Moreover, CX3CR1^+^CD24^-^ macrophages regulated GR1^+^ neutrophils *via* GPR43. Our results demonstrated a unique trilateral relationship among SCFAs, immune cells, and the kidneys during CaOx formation. These findings suggest that future immunotherapies may be used to prevent kidney stones using SCFAs.

## Introduction

Kidney stones are one of the most common urological diseases, with an increasing prevalence and incidence worldwide (1–3). Calcium oxalate (CaOx) stones have, with a high recurrence rate of up to 80%, are the most common type of kidney stones (3, 4); thus, they impose a burden on global public health (1). However, the detailed mechanisms through which internal and environmental factors lead to CaOx stone formation remain unclear (2–5).

CaOx stones arise through crystal nucleation, growth, aggregation, retention, and stone formation (2). Crystal deposits are often accompanied by tissue injury, inflammation, and tissue remodeling (6–8). Recent findings have indicated that crystals trigger inflammation through NLRP3-mediated IL-1β secretion (6, 9, 10). The results of human and animal genome studies have shown that CaOx stones are associated with genes related to oxidative stress, inflammation, immunity, and complement activation pathways (11, 12), which enhance the recruitment of various immune cell subtypes, such as macrophages, dendritic cells (DCs), and T cells (13–16). To date, limited efforts have been exerted to comprehensively understand the landscape of immune cell populations in kidneys with CaOx stones. In recent years, human M2 macrophages were reported to have phagocytic ability that enabled them to degrade fragments of CaOx stones (17), which introduced potential immune-based therapies for nephrolithiasis. Therefore, there is an urgent need to understand the immune-based mechanisms underlying CaOx stone development and to identify more efficient targets for therapeutic treatment.

Previously, we found that short-chain fatty acids (SCFAs) were associated with renal CaOx stone disease (18). SCFAs, the main metabolites produced by gut microbiota fermentation of dietary fiber, play an important role in immunomodulation (19–21). Ohno and Rudensky et al. found that butyrate can regulate the differentiation of regulatory T cells, as well as propionate levels (22, 23). Fachi et al. reported that acetate promotes host innate responses to *Clostridium difficile* through coordination with neutrophils and innate lymphoid cells (20). SCFAs function as ligands for different G protein-coupled receptors through different immune cells (24). However, little is known regarding the immunomodulatory role of SCFAs in CaOx stones.

In this study, we performed mass cytometry (CyTOF) and RNA sequencing on renal immune cells with CaOx crystals to elucidate the landscape of the immune cell populations of kidneys with CaOx stones and then explored the role of SCFAs in the formation of renal CaOx stones through immunomodulatory effects. Understanding the crosstalk among immunity, SCFAs, and kidneys will help define the underlying mechanisms and potential novel therapies for CaOx stones.

## Materials And Methods

### Experimental Animal Studies

C57BL/6J mice were procured from Chengdu Dossy Experimental Animals Co., Ltd. The mice were housed in individual ventilated cages with unlimited access to sterilized food and water. The animal studies were approved by the Animal Research Ethics Committee of the West China Hospital in Sichuan University (2017105A).

Eight-week-old male mice received a single intraperitoneal injection of glyoxylate (80 mg/kg body weight; Sigma-Aldrich, Shanghai, China) for 7 consecutive days (CaOx group) (25, 26). The acetate (C2), propionate (C3), and butyrate (C4) groups received intraperitoneal injections of glyoxylate (80 mg/kg body weight) with 150 mM sodium acetate, sodium propionate, or sodium butyrate, respectively. To deplete the gut microbiota, antibiotic cocktails (Abx), combined with ampicillin (0.5 g/L), vancomycin (0.25 g/L), neomycin (0.5 g/L), and metronidazole (0.5 g/L), were added to the drinking water two weeks before glyoxylate and SCFA administration (Abx, Abx+C2, Abx+C3, and Abx+C4 groups, respectively; n = 8 mice/group). The blood, kidney, and cecal contents of the mice in each group were determined on day 8.

### Adeno-Associated Virus (AAV) Infection

To knockdown GPR43 expression *in vivo*, mice were transduced with an AAV serotype 9 vector encoding a green fluorescent protein reporter together with either short hairpin RNAs (shRNAs) targeting GPR43 in the kidney (AAV-shGPR43) or an empty vector (AAV-null). The shRNA sequences targeting the mouse GPR43 gene were cloned into the AAV by Shanghai GeneChem Co., Ltd. (Shanghai, China). Mice were injected with either AAV-shGPR43 or AAV-null *via* the tail vein (1 × 10^11^ viral particles/mouse), followed by an additional 4 weeks of glyoxylate injection. The mice were randomly divided into five groups and treated with the AAV-null vector (AAV-null group), the AAV-shGPR43 vector and glyoxylate injection (AAV-shGPR43 group), the AAV-shGPR43 vector with C2 in the drinking water (AAV-shGPR43+C2 group), the AAV-shGPR43 vector with C3 in the drinking water (AAV-shGPR43+C3 group), or AAV-shGPR43 vector with C4 in the drinking water (AAV-shGPR43+C4 group).

### CyTOF and Data Analysis

A panel of CyTOF antibodies combined with 42 metal isotope-tagged antibodies (**Supplemental Table 1**, Fluidigm, San Francisco, USA) was used to detect immune cell populations in mouse kidneys. The kidneys were placed in Dulbecco’s modified Eagles’ medium and cut into pieces with collagenase IV (1 mg/mL; Merck, Shanghai, China) and DNase I (100 μg/mL, Merck) at 37°C for 30 min (27). The digested kidney cells were resuspended in phosphate-buffered saline (Thermo Scientific, Shanghai, China) containing 0.02% NaN_3_ and 0.5% bovine serum albumin and stained for viability with 194Pt (Fluidigm) for 5 min on ice. The cells were then blocked using Fc receptors, followed by cell-surface staining with metal-labeled monoclonal antibodies (mAbs) for 30 min. The cells were incubated overnight at 4°C with DNA Intercalator-Ir (Fluidigm) to discriminate singly nucleated cells from doublets. After fixation and permeabilization, the cells were intracellularly stained with metal-labeled mAbs for another 30 min. The cells were washed and prepared for data acquisition. The Helios system (Fluidigm Sciences) was used to acquire CyTOF data at ≤500 events/s.

All CyTOF files were analyzed using FlowJo software, version 10.5.3. (TreeStar, Ashland, OR, USA). The CD45-positive populations were clustered using the X-shift algorithm (28), which automatically pools cells to identify underlying immune cell subpopulations. The normalized expression levels of markers were visualized with a heatmap. Cell frequencies were calculated by dividing the total number of CD45^+^ cells events by the number of assigned cell events. The R software package was used to perform dimension reduction, based on t-distributed stochastic neighbor embedding (t-SNE) (29).

### RNA Sequencing and Analysis

Kidney digestion is the same as described above. Renal cells were surface-stained with fluorophore-conjugated antibodies against the mouse CD45 antigen (FITC, BioLegend, San Diego, CA, USA), then renal CD45^+^ cells were sorted by Flow cytometry. The RNeasy Mini Kit (Qiagen, Germany) was used to extract total RNA from renal CD45^+^ cells following the manufacturer’s instructions. One microgram total RNA was used to prepare an RNA-seq transcriptome library using the TruSeq™ RNA Sample Preparation Kit (Illumina, San Diego, CA). Then, complementary DNA target fragments (200–300 base pairs) were selected, followed by polymerase chain reaction-based amplification with Phusion DNA polymerase (Thermo Scientific, Shanghai, China). An Illumina HiSeq X instrument was used for sequencing.

The raw data were trimmed and qualified using the SeqPrep and Sickle tools. Then, the clean data were aligned to the reference genome using TopHat software (version 2.0.0) (30), and the mapped reads were assembled using StringTie.

Transcript expression levels were calculated using the transcripts per million reads method to identify differentially expressed genes (DEGs) between groups. The RSEM (31) and EdgeR (32) software programs were utilized to quantify gene abundances and for differential expression analysis, respectively. Furthermore, Gene Ontology (GO) functional-enrichment analysis and Kyoto Encyclopedia of Genes and Genomes (KEGG) pathway analysis were performed on DEGs with a Bonferroni-corrected P-value of ≤ 0.05.

### Flow Cytometry

Isolated kidney CD45^+^ cells were used to analyze immune subtypes. The cells were surface-stained with fluorophore-conjugated antibodies against the following mouse antigens: CD45 (FITC, APC), CD11b (PE), CD11c (FITC), F4/80 (PE/CY7), CX3CR1 (BV510), CD24 (APC), GR-1 (APC), I-A/I-E (APC/CY7), and CD103 (BV421) (BioLegend, San Diego, CA, USA). A FACSAria SORP flow cytometer (BD Biosciences, CA, USA) and FlowJo software were used to acquire and analyze the flow cytometric data, respectively.

### Histological Detection and Immunofluorescence

Kidneys were fixed in paraformaldehyde, embedded in paraffin, and cut into 4 µm sections. Hematoxylin and eosin staining and von Kossa staining were performed using staining kits (Solarbio, China), according to the manufacturer’s protocols. For immunofluorescence, the sections or cells were incubated with an anti-F4/80/Alexa Fluor^®^ 647-conjugated antibody (1:100, BioLegend), an anti-GPR43/FITC-conjugated antibody (1:100, Bioss, Beijing, China), an anti-CX3CR1/PE-conjugated antibody (1:100, Novus, CO, USA), and an anti-GR-1/Alexa Fluor^®^ 594-conjugated antibody (1:100, BioLegend), and with DAPI (Merck) or Hoechst (Thermo Scientific, Shanghai, China) dye.

### Cytokine Analyses

Mouse sera were collected, and IL-1β, IL-6, and TNF-α levels were measured using enzyme-linked immunosorbent assays (Invitrogen, Shanghai, China), according to the manufacturer’s recommendations.

### 16S rRNA Microbial-Profiling Analysis

An E.Z.N.A.^®^ Soil DNA Kit (Omega Bio-tek, Inc., GA, USA) was used to extract microbial DNA from cecal samples. According to our previous study, primers 338F (5’-ACTCCTACGGGAGGCAGCAG-3’) and 806R (5’-GGACTACHVGGGTWTCTAAT-3’) were used to amplify the bacterial 16S rRNA genes (V3–V4 hypervariable regions) (18). Sequencing was performed by Majorbio Technology Co. Ltd. (Shanghai, China) using the Illumina MiSeq PE300 platform (Illumina).

The raw 16S rRNA sequence data were demultiplexed, quality-filtered, and merged using fastp software (version 0.20.0) (33) and FLASH software (version 1.2.7) (34). All sequences were clustered into different operational taxonomic units (OTUs) at 97% similarity using UPARSE software (version 7.1) (35) by removing the chimeric sequences. The taxonomy of the 16S rRNA amplicon sequences was analyzed using the RDP Classifier software (version 2.2) (36) against the Silva v138 database with a confidence threshold of 0.7.

Alpha diversity and coverage indices were calculated. Principal coordinates analysis (PCoA) and the Adonis test were used to evaluate beta diversity among the groups. Linear discriminant analysis effect size (LEfSe) analysis was used to identify differentially abundant bacteria among the three groups with a cutoff threshold of 3.0. KEGG metabolic pathways were predicted using the PICRUSt software package.

### Cell Coculture Assay

Mouse TCMK-1 kidney epithelial cells (3 × 10^4^) were treated with lipopolysaccharide (LPS, 1 µg/ml) in a 24-well plate and cocultured with or without acetate (C2), propionate (C3), butyrate (C4), or immune cells for 24 h. Then, CaOx crystals (100 µg/ml) were added to the coculture system for 10 min, followed by fixation. Crystal adhesion to the cell surface was observed using an inverted microscope.

CD45^+^F4/80^+^CD11b^+^CX3CR1^+^CD24^-^ macrophages and CD45^+^CD11b^+^GR1^+^ neutrophils were sorted by flow cytometry. Macrophages (with or without GPR43-antagonist treatment for 24 h) were cocultured with neutrophils for another 24 h. GPR43 expression was detected by immunofluorescence. Using a Transwell system, macrophages were cultured in the upper chamber with or without a GPR43 antagonist for 24 h. Treated macrophages were added to another upper chamber of a 24-well plate with neutrophils, and TCMK-1 cells were treated with LPS and CaOx crystals seeded in the lower chamber for 24 h. Neutrophil migration and GPR43 expression were also observed.

### Statistics

Statistical analyses were performed using SPSS. Data are shown as mean ± standard deviation (SD). All data were analyzed by one-way analysis of variance for multiple groups, and Student’s t-test was applied for comparisons between two groups. Statistically significant differences are shown as *p < 0.05 or **p < 0.01.

## Results

### Phenotypic Heterogeneity Within the Kidney-Resident Immune Cell Population

After 7 consecutive days of intraperitoneal glyoxylate injection, CaOx crystal deposits were found in mouse kidneys with elevated serum creatinine and urea levels (**Supplemental Figure 1**). To explore the immunomodulatory role of SCFAs in CaOx stones, we first studied the phenotypic diversity of immune cell populations in kidneys with CaOx crystals by analyzing CD45^+^ cell populations clustered with the X-shift algorithm.

**Figure 1:**
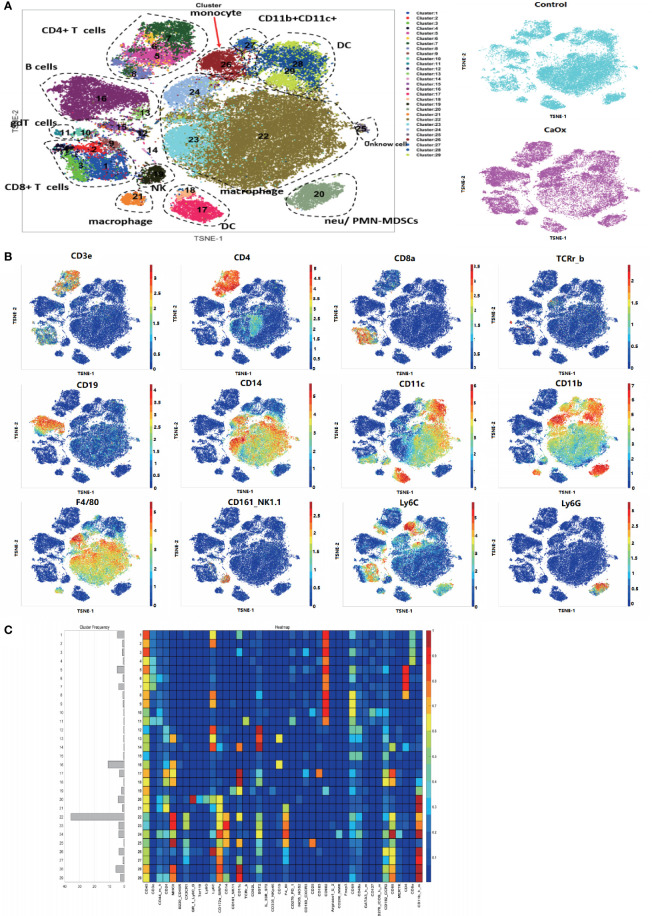
Identification of immune cell populations in kidney using mass cytometry. **(A)** tSNE visualization of 383,985 renal CD45^+^ cells merged events from control mice and CaOx mice. **(B)** Feature plots shows the canonical markers of immune cells including CD3, CD8, CD4, CD19, CD14, CD11c, CD11b, F4/80, NK1.1, Ly6C, and Ly6G. **(C)** Heatmap of different markers in different clusters.

Unsupervised clustering of 383,985 single CD45^+^ kidney cells from six samples created a detailed t-SNE map of distinct cell populations (**Figure 1A**). The clusters were classified into 29 distinct immune cell types (**Figures 1A, B** **)**, based on the expression levels of lineage markers. The normalized expression of clusters is shown in a heatmap (**Figure 1C**). We identified the following major cell subsets: CD4 T cells, CD8 T cells, B cells, macrophages, DCs, natural killer cells, monocytes, ILCs, and neutrophils (**Supplemental Table 2**).

**Figure 2:**
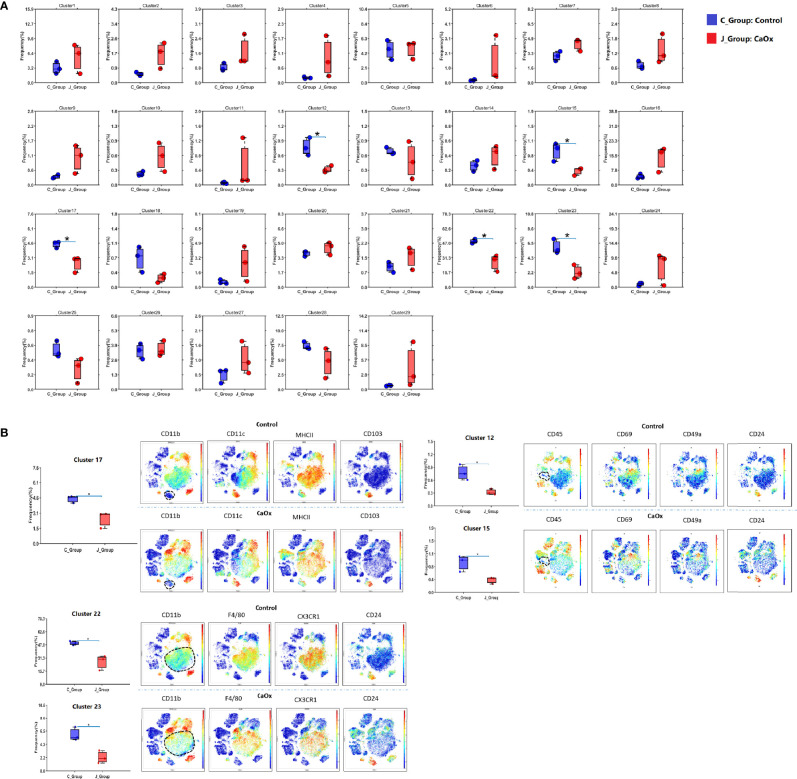
Cluster frequencies in kidney of control mice and CaOx mice. **(A)** Frequencies of 29 clusters between groups. **(B)** Marker of significant differential clusters between groups. C_group stands for control group, and J_Group stands for CaOx group. Statistically signifificant was shown as *p < 0.05.

The frequencies of each cluster are shown in Fig. 2. The frequencies of clusters 12, 15, 17, 22, and 23 in CaOx crystals were significantly lower than those in control mice (**Figure 2A**). Based on lineage and functional markers, the cells of cluster 17 were defined as DCs with high CD103 expression that also expressed CD24, MHC-II, BST2, CXCR3, CD69, CCR2, and CD68 (**Figure 2B**). Clusters 22 and 23 were both defined as CX3CR1^+^CD24^-^ macrophage populations, where CD11c expression differentiated cluster 22 from cluster 23 (**Figure 2B**). Clusters 12 and 15 expressed low levels of CD45, and their cell types could not be determined (**Figure 2B**).

**Figure 3:**
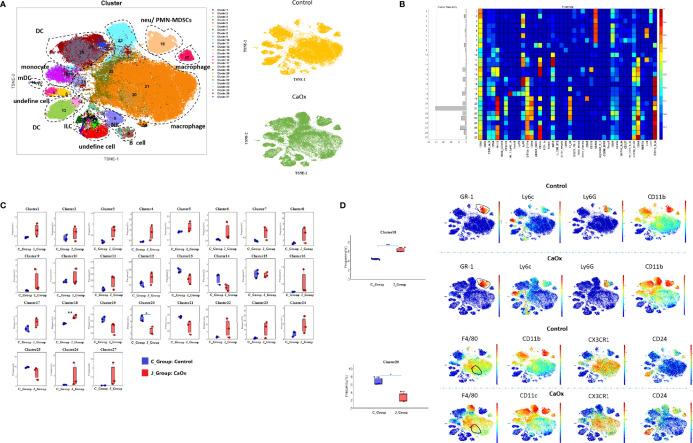
Identifification of Non-CD3+ and Non-CD19+ cell populations in kidney using mass cytometry. **(A)** tSNE visualization of Non-CD3+ and Non-CD19+ cell populations by X-shift algorithm. **(B)** Heatmap of different markers in different clusters. **(C)** Frequencies of 27 clusters between groups. **(D)** Phenotypical characteristics of signifificant differential clusters between control and CaOx mice. Statistically signifificant was shown as *p < 0.05, **p < 0.01.

When analyzing the above results, we did not find a significant difference in the frequencies of T cells and B cells. We then depleted CD3^+^ and CD19^+^ cells to an unsupervised cluster using the X-shift algorithm to identify more myeloid cell subsets. We observed 27 clustered immune cell populations, composed mainly of macrophages and DCs (**Figures 3A, B** **)**. Cluster 18 (defined as neutrophils) increased significantly in mice with CaOx crystals, and cluster 20 (defined as CX3CR1^+^CD24^-^ macrophages) was significantly lower in mice with CaOx crystals than in control mice (**Figures 3C, D** **)**, indicating that macrophages and neutrophils were involved in CaOx crystal formation in the kidney.

### Functional and Transcriptional Features of CD45^+^ Cells in the Kidneys of Mice With CaOx Crystals

**Figure 4:**
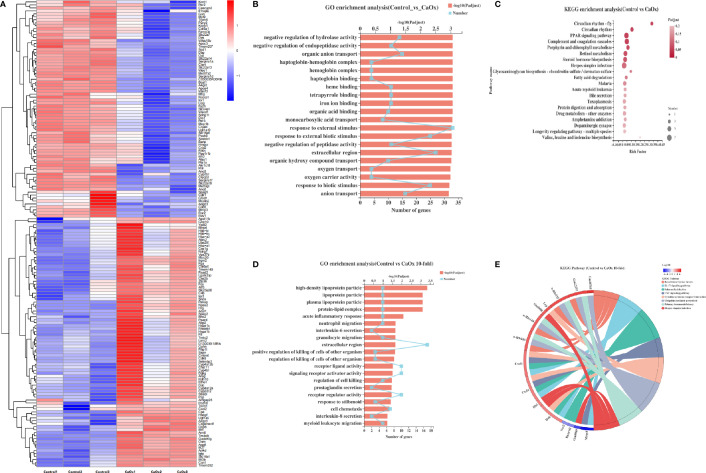
Transcriptional features of CD45^+^ cells in kidney of CaOx crystal mice. **(A)** Differentially expressed genes (DEGs) between control and CaOx mice. **(B)** GO (Gene Ontology) analyses of DEGs (P adjust<0.05, FC≧2 and FC≦0.5). **(C)** KEGG (Kyoto Encyclopedia of Genes and Genomes) analyses of DEGs (P adjust<0.05, FC≧2 and FC≦0.5). **(D)** GO analyses of DEGs (P adjust<0.05, FC≧10 and FC≦0.1). **(E)** KEGG analyses of DEGs (P adjust<0.05, FC≧10 and FC≦0.1), and KEGG pathways invlovled genes (P adjust<0.05, FC≧10 and FC≦0.1).

Bulk RNA sequencing was applied to identify the functional and transcriptional characteristics of immune kidney cells in mice with CaOx crystals. We identified 172 DEGs in mice with CaOx crystals (adjusted P < 0.05; fold-change ≥ 2 or ≤ 0.5), including 89 upregulated and 83 downregulated mRNAs (**Figure 4A**). GO analyses revealed DEGs associated with responses to external stimuli, extracellular regions, and other parameters (**Figure 4B**). KEGG analyses showed that the DEGs were involved in circadian rhythms, the PPAR signaling pathway, complement and coagulation cascades, retinol metabolism, porphyrin and chlorophyll metabolism, and steroid hormone biosynthesis (**Figure 4C**). To further identify the functional and transcriptional features of the more significant DEGs, we analyzed DEGs with 10-fold changes in expression. Interestingly, DEGs with 10-fold expression differences were enriched for GO terms related to acute inflammatory responses, neutrophil and granulocyte migration, and interleukin-6 and interleukin-8 secretion, among others (**Figure 4D**). In addition, KEGG analysis showed that DEGs with 10-fold expression differences were linked to the IL-17 and TNF signaling pathway, and cytokine–cytokine receptor interactions (**Figure 4E**), where most genes were associated with CXCL2, CXCL1, and IFN-γ (**Figure 4E**). Previous findings showed that these genes were all expressed at sites of inflammation and were related to monocytes and neutrophils (37, 38). The results are in accordance with our CyTOF results, suggesting that the formation of kidney stones was associated with inflammation promoted by neutrophil migration.

### SCFAs Reduced the CaOx Crystal Deposition by Regulating Immune Cell Subpopulations and Inflammation

**Figure 5:**
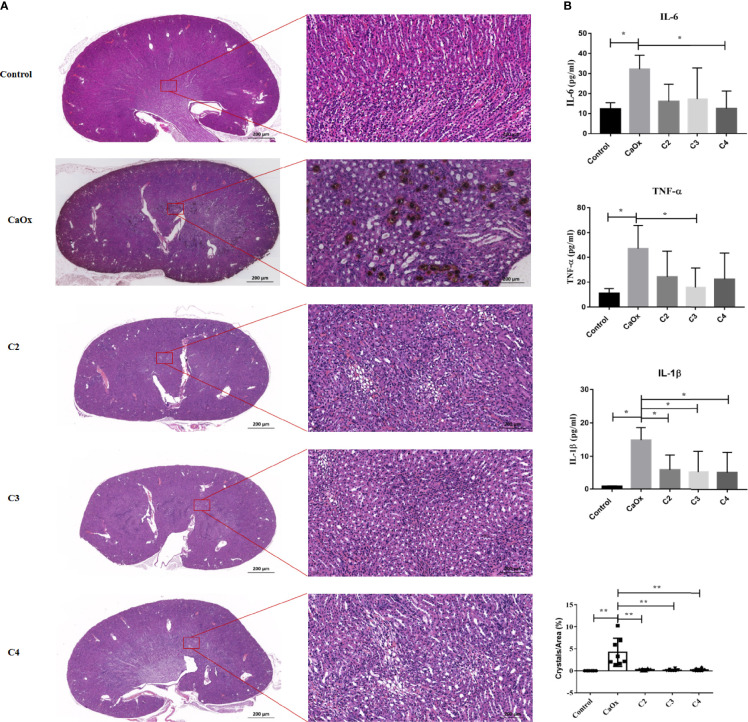
Effect of short chain fatty acid (SCFAs) on renal CaOx crystal formation. **(A)** Representative images of Von Kossa staining and HE staining in kidney of mice administrated with C2(acetate), C3 (propionate) and C4 (butyrate). **(B)** The level of serum IL-1β, IL-6 and TNF-α. Data were shown as mean ± standard deviation. All data were analyzed by one-way ANOVA followed by Tukey’s multiple comparisons test among multiple groups. Statistically signifificant was shown as *p < 0.05, **p < 0.01.

Previously, we found that SCFAs were associated with kidney stones (18), which play an important role in immunomodulation. Thus, we next administered SCFAs to mice with renal CaOx crystals to observe the effect of SCFAs on crystal formation through immunomodulation. The results showed that C2, C3, and C4 reduced CaOx crystal deposition (**Figure 5A**), with decreased secretion of serum IL-6, TNF-α, and IL-1β (**Figure 5B**) and decreased expression of NLRP3 (**Supplemental Figure 2**).

**Figure 6:**
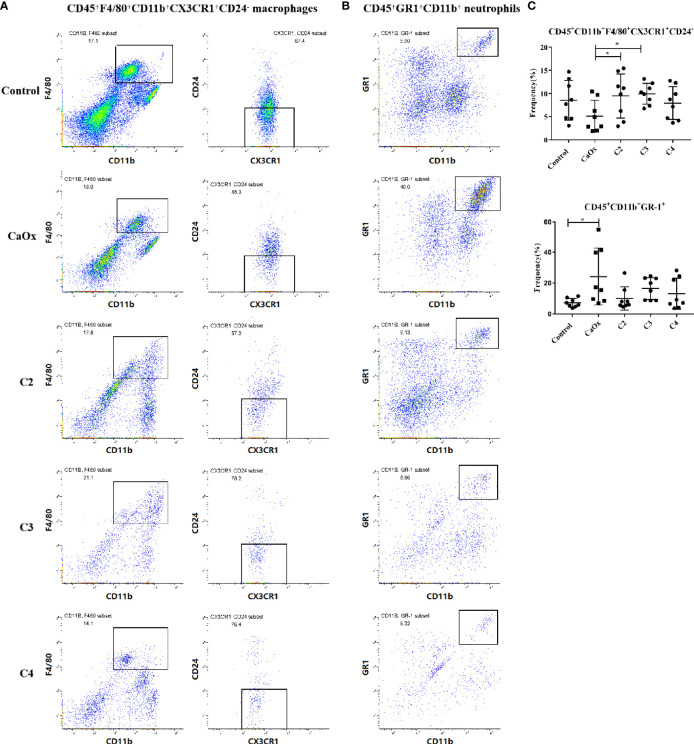
Representative flow cytometric analysis of CD45^+^F4/80^+^CD11b^+^CX3CR1^+^CD24^-^ macrophages and CD45^+^GR1^+^CD11b^+^ neutrophil in CaOx crystal mice with or without administration of SCFAs. Gates were set on CD45^+^ cells, **(A)** macrophages then were gated on F4/80^+^CD11b^+^ cells followed by CX3CR1^+^CD24^-^, and **(B)** neutrophil were gated as GR1^+^CD11b^+^ in CD45^+^ cells. **c** Frequencies of CD45^+^F4/80^+^CD11b^+^CX3CR1^+^CD24^-^ macrophages and CD45^+^GR1^+^CD11b^+^ neutrophil in kidney with or without administration of SCFAs. **(C)** Frequencies of CD45^+^F4/80^+^CD11b^+^CX3CR1^+^CD24^-^ macrophages and CD45^+^GR1^+^CD11b^+^ neutrophil in kidney. Data were shown as mean ± standard deviation. Data were analyzed by one-way ANOVA followed by Tukey’s multiple comparisons test among multiple groups. Statistically signifificant was shown as *p < 0.05.

The frequencies of CD45^+^F4/80^+^CD11b^+^CX3CR1^+^CD24^-^ macrophages (CX3CR1^+^CD24^-^ macrophages) and CD45^+^GR1^+^CD11b^+^ neutrophils (GR1^+^ neutrophils) in mice with CaOx crystals were detected by flow cytometry (**Supplemental Figure 3**). In mice with renal CaOx crystals, CX3CR1^+^CD24^-^ macrophages decreased, and GR1^+^ neutrophils increased significantly (**Figures 6A, B** **)**. Interestingly, we found that C2 and C3 administration significantly increased the frequencies of CX3CR1^+^CD24^-^ macrophages (**Figure 6C**). Administration of C2, C3, and C4 decreased the frequency of GR1^+^ neutrophils (**Figure 6C**). These results suggest that SCFAs prevent renal CaOx crystal formation through immunomodulation.

### Immunomodulation of Renal CaOx Crystal Formation in Mice by SCFAs Depended on the Gut Microbiota

**Figure 7:**
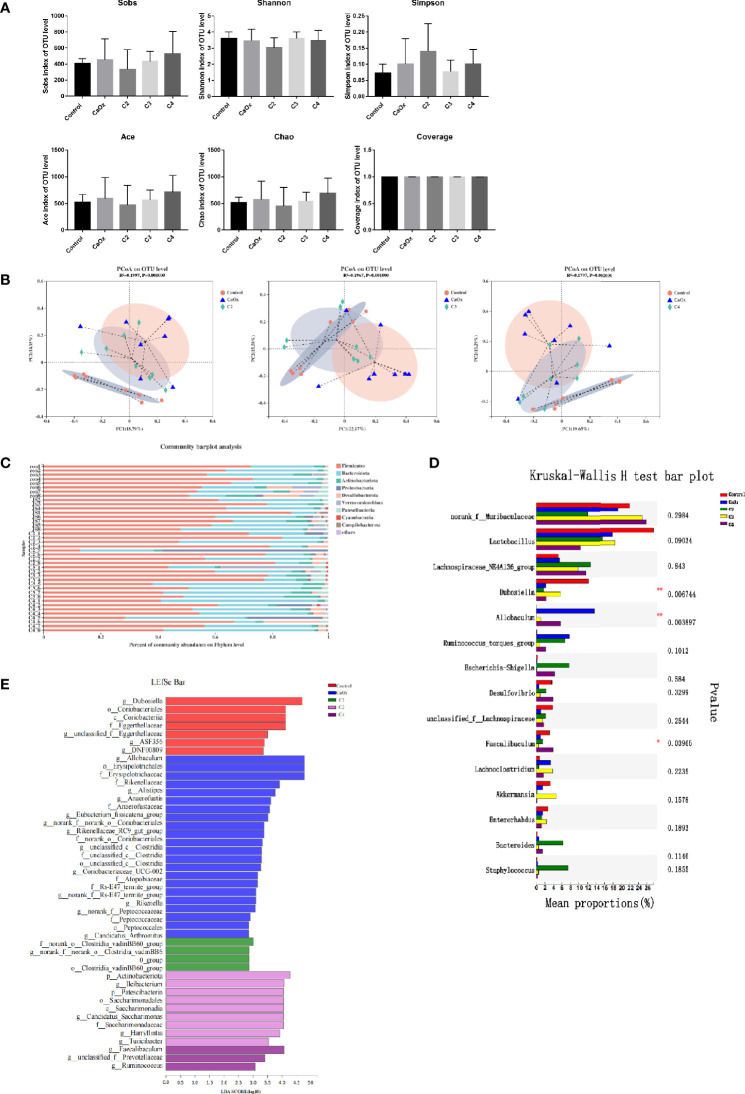
Analysis of caecal microbiota among controls, CaOx mice and mice administration of SCFAs by using 16s rRNA. **(A)** Comparison of alpha diversity of gut microbiota among controls, CaOx mice and mice administration of C2, C3 and C4. Sobs, Shannon, Simpson, Ace, Chao, and Coverage indices at OTUs level were compared among groups by the Wilcoxon rank-sum test. **(B)** Comparison of beta diversity of gut microbiota among controls, CaOx mice and mice administration of C2, C3 and C4. PCoA score plot based on binary-pearson distance at OTUs level revealed classification of different groups. **(C)** The composition of gut microbiota in controls, CaOx mice and mice administration of C2, C3 and C4 at phylum level. **(D)** Different bacteria among groups using Kruskal-Wallis H test, *p < 0.05, **p < 0.01. **(E)** Bacteria with higher relative abundance in the five groups by LEfSe analysis.

To determine whether SCFAs affected the composition of the gut microbiota, caecum microbiota were detected after SCFA treatment in mice. All sequences were clustered into 2199 OTUs belonging to 767 genera and 47 phyla. The coverage indices were greater than 98%. The Wilcoxon rank-sum test showed that alpha diversity indices (Sobs, Simpson, Shannon, Ace, Chao, and Coverage) were not significantly different among the groups (**Figure 7A**). PCoA at the OTU level showed that the microbiota composition of the control mice, CaOx crystal mice, and CaOx crystal mice administered with C2, C3, and C4 were different, which was confirmed by the Adonis test (*p < 0.01*) (**Figure 7B**).

At the phylum level, Firmicutes was the most common bacteria in the gut microbiota, followed by Bacteroidetes and Actinobacteria (**Figure 7C**). At the genus level, we found that the abundance of Allobaculum was highest in mice with CaOx crystals, although the abundance decreased sharply in mice with CaOx crystals that were administered C2 and C3 (*p < 0.01*, **Figure 7D**). The abundances of Dubosiella, Candidatus, Saccharimonas, and Bifidobacterium significantly increased in mice with CaOx crystals administered C3 (*p < 0.05*, **Figure 7D**). Faecalibaculum was significantly increased in mice with CaOx crystals administered C4 (**Figure 7D**). LEfSe analysis significantly different abundances (*p < 0.05*) for six, ten, three, six, and 18 bacterial genera in the gut microbiota of mice with CaOx crystals administered C2, C3, and C4; control mice; and CaOx crystal mice, respectively (**Figure 7E** and **Supplemental Table 3**). The differentially abundant metabolic pathways are shown in **Supplemental Table 4**. PICRUSt analysis revealed that the abundance of 24 KEGG pathways differed significantly among the five groups, including those related to oxidative phosphorylation; fructose and mannose metabolism; glycerophospholipid metabolism; and valine, leucine, and isoleucine degradation (*p < 0.05*).

**Figure 8:**
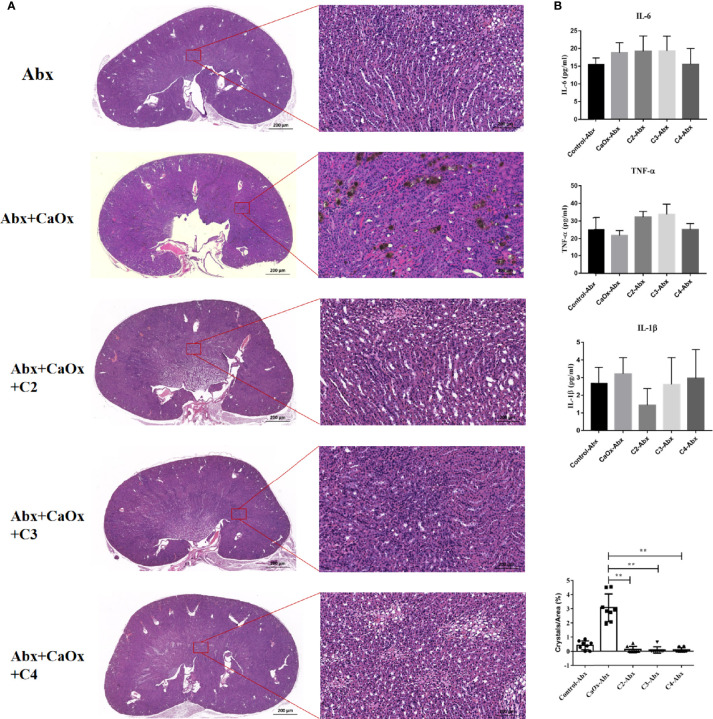
Effect of antibiotics on renal CaOx crystal formation in mice with or without SCFAs. **(A)** Representative images of Von Kossa staining and HE staining in kidney of mice administrated with antibiotics and C2(acetate), C3 (propionate) and C4 (butyrate), respectively. **(B)** The level of serum IL-1β, IL-6 and TNF-α. Data were shown as mean ± standard deviation. All data were analyzed by one-way ANOVA followed by Tukey’s multiple comparisons test among multiple groups. Statistically signifificant was shown as **p < 0.01.

**Figure 9:**
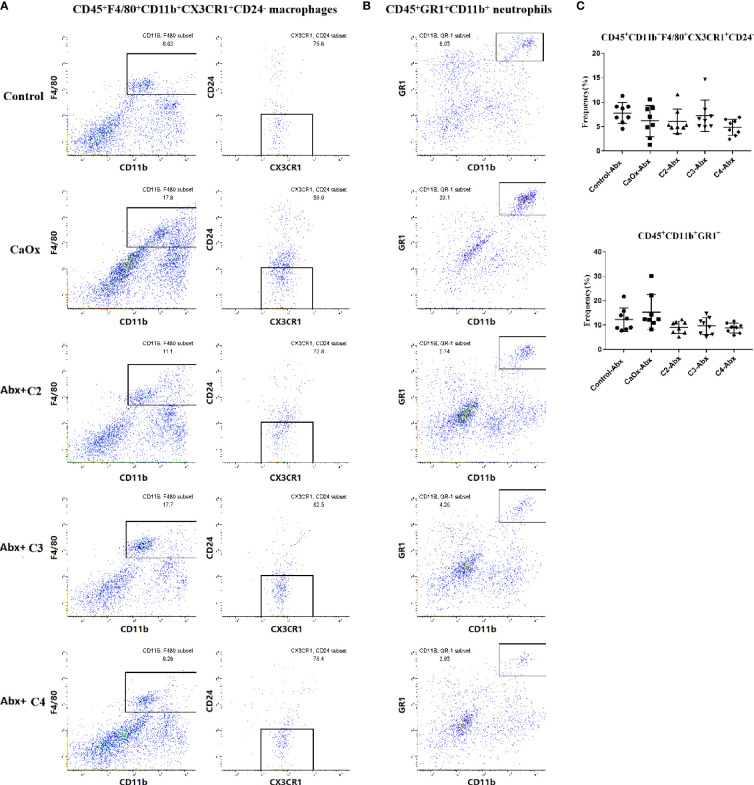
Representative flow cytometric analysis of CD45^+^F4/80^+^CD11b^+^CX3CR1^+^CD24^-^ macrophages and CD45^+^GR1^+^CD11b^+^ neutrophil in CaOx crystal mice with administration of SCFAs after treatment of antibiotics. **(A)** macrophages were first gated on CD45^+^ cells, and then were gated on F4/80^+^CD11b^+^ cells followed by CX3CR1^+^CD24^-^, and **(B)** neutrophil were gated as GR1^+^CD11b^+^ in CD45^+^ cells. **c** Frequencies of CD45^+^F4/80^+^CD11b^+^CX3CR1^+^CD24^-^ macrophages and CD45^+^GR1^+^CD11b^+^ neutrophil in kidney with administration of SCFAs and antibiotics. **(C)** Frequencies of CD45^+^F4/80^+^CD11b^+^CX3CR1^+^CD24^-^ macrophages and CD45^+^GR1^+^CD11b^+^ neutrophil in kidney. Data were shown as mean ± standard deviation. Data were analyzed by one-way ANOVA followed by Tukey’s multiple comparisons test among multiple groups.

To determine whether SCFAs regulated the formation of CaOx crystals in a manner dependent on the gut microbiota, antibiotics were administered to deplete intestinal bacterial loads before SCFAs were used. Administering C2, C3, and C4 with antibiotics reduced CaOx crystal deposition (**Figure 8A**), without decreasing secretion of IL-6, TNF-α, and IL-1β (**Figure 8B**). Interestingly, we found that administration of C2, C3, and C4 did not modulate the frequencies of CX3CR1^+^CD24^-^ macrophages and GR1^+^ neutrophils (**Figure 9**) after depletion of the intestinal bacterial load. These results suggest that SCFAs prevent renal CaOx crystal formation, although the immunomodulatory effects depend on the gut microbiota.

### SCFAs Alleviated Crystal Formation and Adhesion to Kidney Cells by Regulating Immune Cells With GPR43

**Figure 10:**
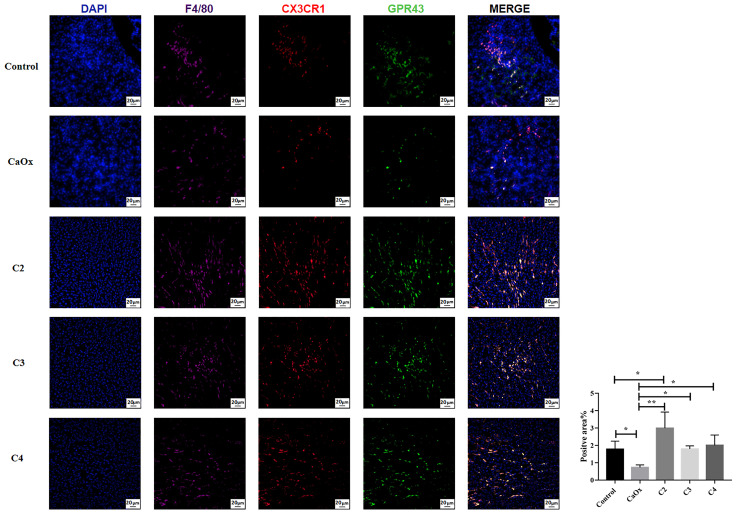
Representative images of immunofluorescence staining of macrophages in kidney of controls, CaOx mice and CaOx mice administration of SCFAs. 647 (F4/80, Violet), PE (CX3CR1, red) and FITC (GPR43, green) fluorescence images and merged images with DAPI staining (blue) of the same sections are also shown. Statistically signifificant was shown as *p < 0.05, **p < 0.01.

**Figure 11:**
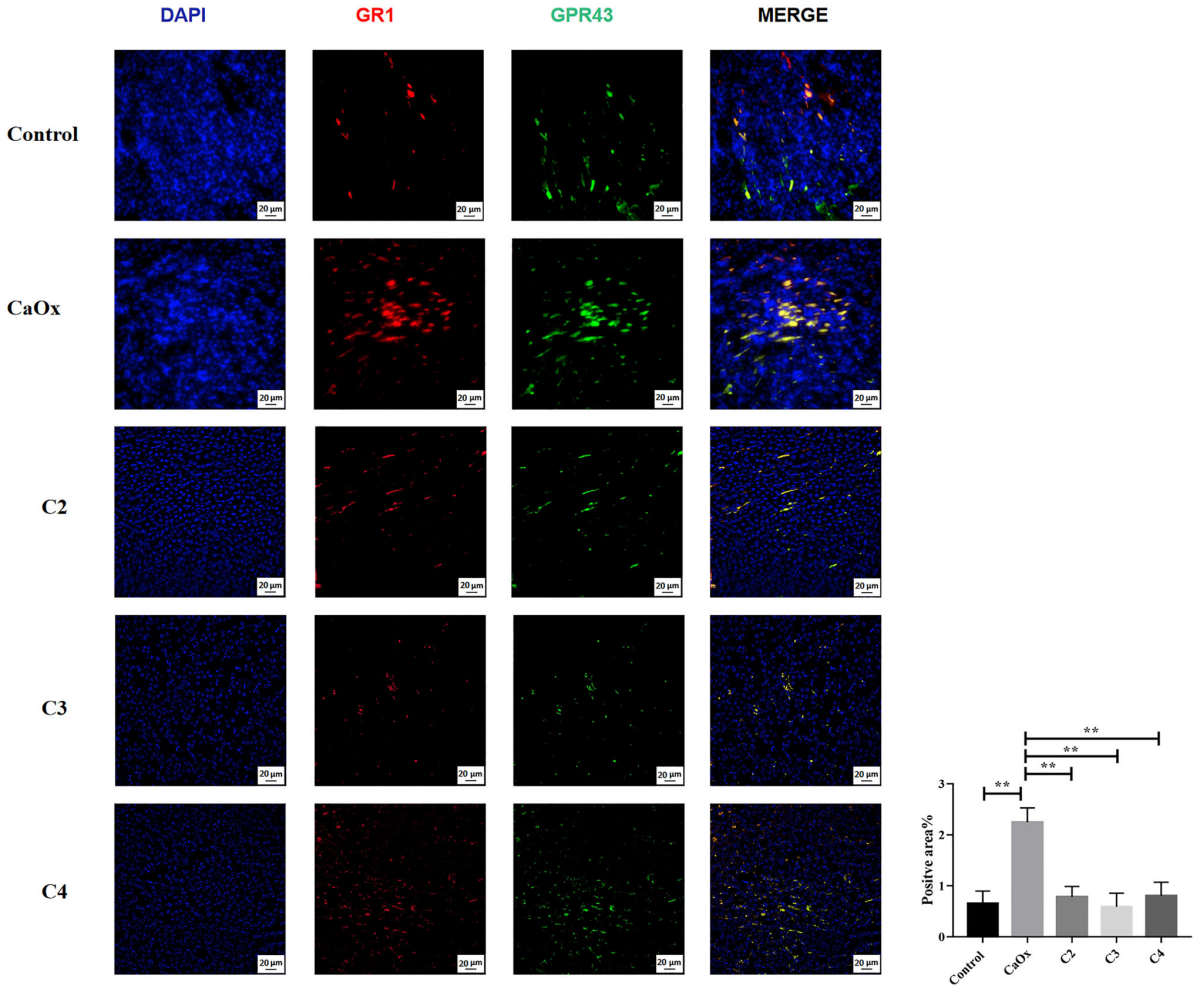
Representative images of immunofluorescence staining of neutrophils in kidney of controls, CaOx mice and CaOx mice administration of SCFAs. 594 (GR1, red), and FITC (GPR43, green) fluorescence images and merged images with DAPI staining (blue) of the same sections are also shown. Statistically signifificant was shown as **p < 0.01.

GPR43, known as SCFA receptors, is activated by SCFAs (39) and has been reported to be expressed on extensive types of immune cells, particularly neutrophils and monocytes (40, 41). To identify the role of GPR43 in SCFAs in regulating crystal formation through kidney immune cells, GPR43 was detected in the kidneys of mice with or without SCFA administration. GPR43 expression on CX3CR1^+^CD24^-^ macrophages in the kidneys of mice with CaOx crystals was lower than that in control mice (**Figure 10**). C2, C3, and C4 administration increased the infiltration of CX3CR1^+^CD24^-^GPR43^+^ macrophages with GPR43 (**Figure 10**). In contrast, GPR43 expression on GR1^+^ neutrophils in the kidney decreased after SCFA administration, compared to that in mice with CaOx crystals (**Figure 11**).

**Figure 12:**
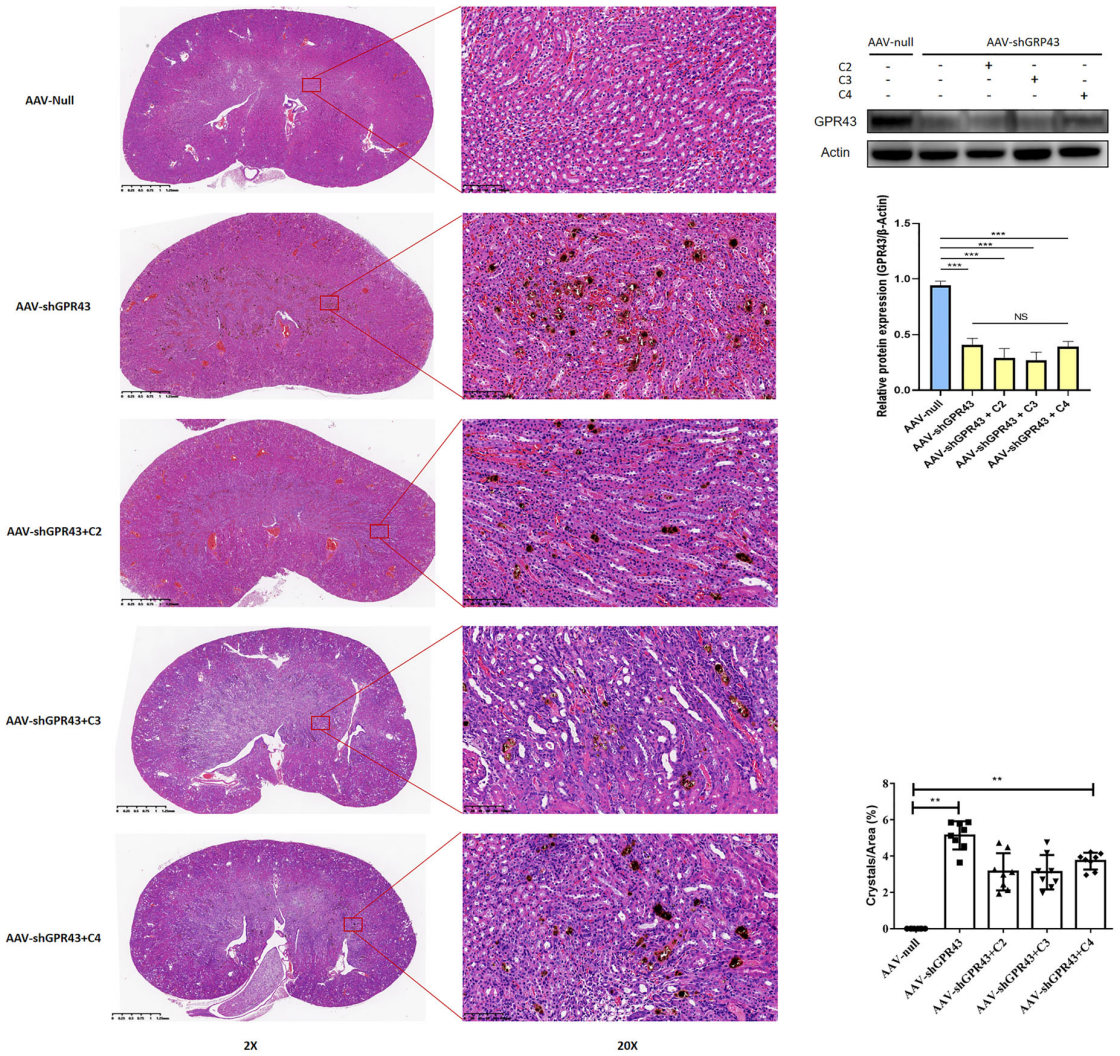
Representative images of Von Kossa staining and HE staining in kidney after administration of AAV carrying shRNA targeting GPR43. AAV-shGPR43 represent as groups of mice treated with AAV carrying shRNA targeting GPR43 and administration with SCFAs. Western Blot was used to detect GPR43 expression on protein level. Statistically significant was shown as **p < 0.01, ***p < 0.001.

Then, AAV carrying GPR43 shRNA was administered to mice with CaOx crystals, along with SCFAs. Renal GPR43 expression was knocked down by transduction with the AAV vector after four weeks (**Figure 12**). GPR43 knockdown inhibited the alleviation of crystal formation in the kidney by SCFAs (**Figure 12**). We also found that GPR43 knockdown blocked the regulation of CX3CR1^+^CD24^-^ macrophages and GR1^+^ neutrophils by SCFAs (**Supplemental Figure 4**).

**Figure 13:**
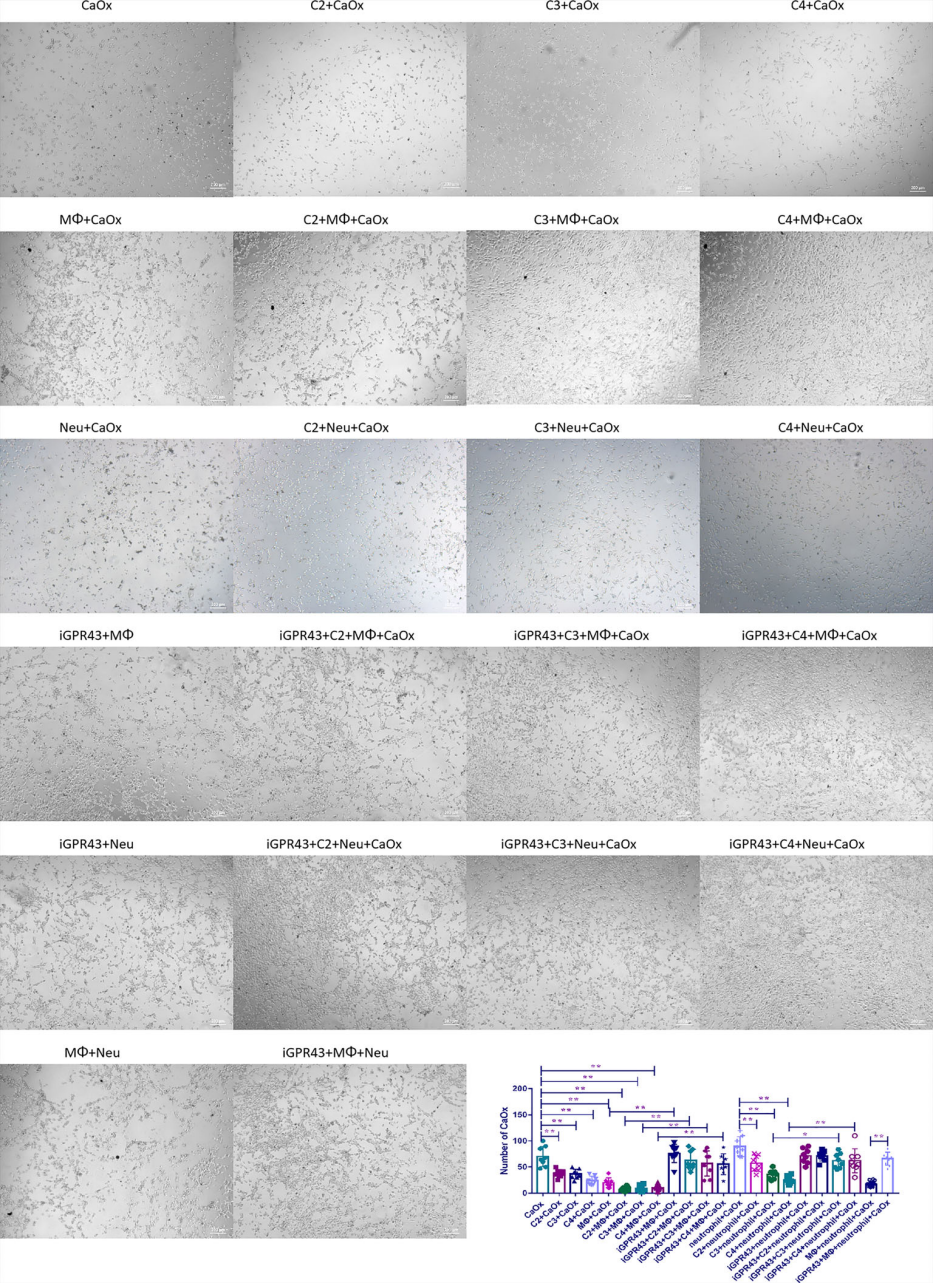
Adhesion of CaOx crystals on mouse renal tubular epithelial cells (TCMK-1 cells) when treated with SCFAs, macrophages, neutrophils and GPR43 antagonist. Statistically significant was shown as *p < 0.05, **p < 0.01.

To evaluate the effect of SCFAs on immune cells *via* GPR43, SCFAs and GPR43 antagonists (with or without immune cells) were added to mouse renal tubular epithelial cells (TCMK-1 cells) and a CaOx crystal coculture system *in vitro*. C2, C3, and C4 treatment reduced CaOx crystal adhesion to TCMK-1 cells. CX3CR1^+^CD24^-^ macrophages showed decreased adhesion to CaOx crystals on TCMK-1 cells, but GR1^+^ neutrophils showed increased adhesion to CaOx crystals, when compared to the CaOx group (**Figure 13**). After coculturing with SCFAs, CX3CR1^+^CD24^-^ macrophages showed enhanced adhesion to CaOx crystals on TCMK-1 cells, which was reversed by the GPR43 antagonist (**Figure 13**). GR1^+^ neutrophils showed decreased adhesion CaOx crystals on TCMK-1 cells after coculture with SCFAs; however, the GPR43 antagonists enhanced CaOx crystal adhesion to TCMK-1 cells when cocultured with GR1^+^ neutrophils and SCFAs (**Figure 13**). These results suggest that SCFAs not only alleviate crystal adhesion to renal cells, but also play a role in regulating immune cells *via* GPR43.

**Figure 14:**
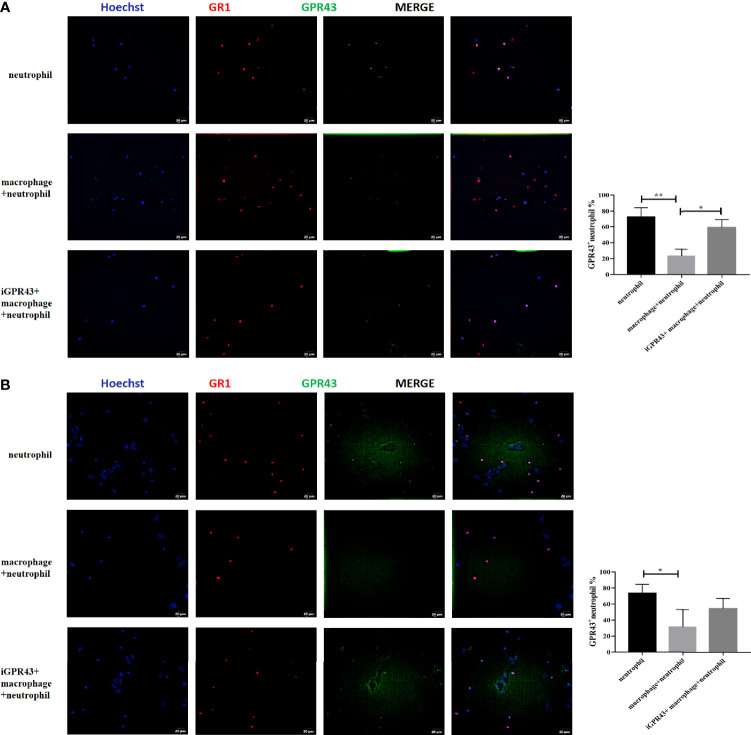
Representative images of immunofluorescence staining of neutrophils and GPR43. **(A)** Macrophages were cocultured with neutrophils in non-transwell system with or without GPR43 antagonist. **(B)** Macrophages were cocultured with neutrophils in the upper chamber of transwell system with or without GPR43 antagonist. TCMK-1 cells were stimulated with CaOx crystals and LPS in the lower chamber of transwell system. 594 (GR1, red), and FITC (GPR43, green) fluorescence images and merged images with Hoechst staining (blue) of the same visual field are also shown. Statistically significant was shown as *p < 0.05, **p < 0.01.

In addition, we found that CX3CR1^+^CD24^-^ macrophages inhibited CaOx crystal adhesion to TCMK-1 cells induced by GR1^+^ neutrophils through GPR43 (**Figure 13**). Next, we cocultured CX3CR1^+^CD24^-^ macrophages and GR1^+^ neutrophils in a non-Transwell or a Transwell system, with or without TCMK-1 cells or CaOx crystals. CX3CR1^+^CD24^-^ macrophages suppressed GPR43 expression on GR1^+^ neutrophils (**Figure 14A**) and decreased the migration of GR1^+^ neutrophils (**Figure 14B**). However, treating CX3CR1^+^CD24^-^ macrophages with GPR43 antagonists restored GPR43 expression on neutrophils and the migration of GR1^+^ neutrophils (**Figure 14**). The results indicate that the interaction between CX3CR1^+^CD24^-^ macrophages and GR1^+^ neutrophils was mediated through GPR43.

**Figure 15:**
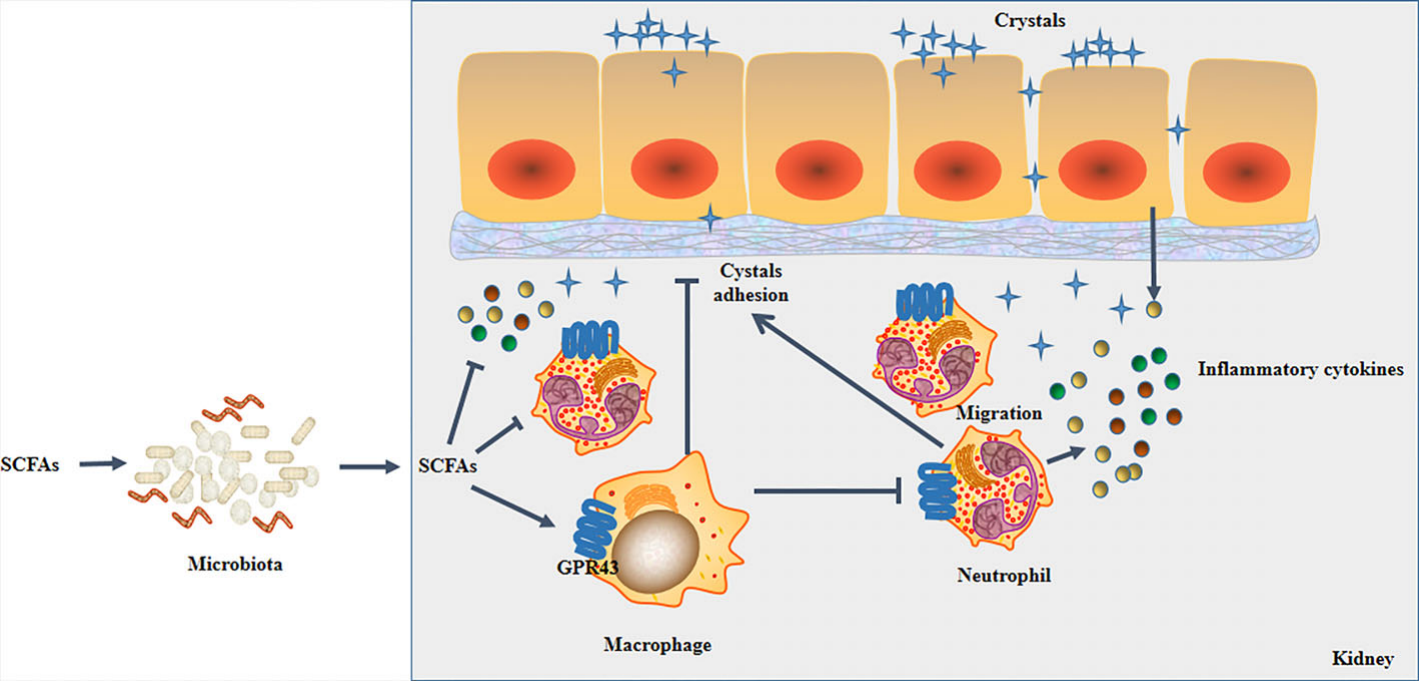
Schematic overview of SCFAs on renal CaOx crystal formation through immunomodulation.

In summary, we found multiple immune cell subpopulations in the kidney. We and also found that SCFAs reduced CaOx crystal formation in the kidney by decreasing the secretion of IL-1β, IL-6, and TNF-α by modulating CX3CR1^+^CD24^-^ macrophages and GR1^+^ neutrophils in a manner that depended on the gut microbiota. Furthermore, SCFAs alleviated crystal formation and crystal adhesion in the kidney by regulating macrophages and neutrophils with GPR43. Additionally, our results revealed that CX3CR1^+^CD24^-^ macrophages regulate GR1^+^ neutrophils through GPR43 (**Figure 15**).

## Discussion

In this study, we first applied CyTOF and mRNA sequencing with kidney immune cells with CaOx crystals to elucidate the characteristics of immune populations in kidneys with CaOx crystals. We then explored the immunomodulatory role of SCFAs in the kidneys of mice with CaOx. Recently, several groups reported that macrophages play an important role in kidney stone disease. Sergei et al. found that macrophages removed and digested interstitial renal crystal deposits by releasing inflammatory cytokines (42). Further data shown that inflammatory macrophages (M1) were associated with stone formation and that anti-inflammatory macrophages (M2) were associated with the suppression of stone formation (42). In this study, five subsets of macrophages were identified in mouse kidneys, including SIRPα+ macrophages, CD11c+CX3CR1+ macrophages, CD11c-CX3CR1+ macrophages, and M2 and CD25+ macrophages. However, the abundances of CD11c+CX3CR1+ macrophages and CD11c-CX3CR1+ macrophages, but not M2 macrophages, were significantly reduced in kidneys with CaOx crystals. CX3CR1, a chemokine receptor for monocytes/macrophages, plays an important role in acute injury repair by regulating macrophage phagocytosis and cytokine production (43). Marelli et al. showed that macrophages expressing CX3CR1 contribute to maintaining the inflammatory response balance in the gut by secreting IL-10 (44). In this study, CX3CR1^+^CD24^-^ macrophages were cocultured with TCMK-1 cells, and we found that CX3CR1^+^CD24^-^ macrophages decreased the adhesion of CaOx crystals to TCMK-1 cells and inhibited CaOx crystal adhesion to TCMK-1 cells induced by neutrophils. These results suggest that CX3CR1^+^CD24^-^ macrophages play an anti-inflammatory role in kidneys with CaOx crystals.

Neutrophils and neutrophil extracellular traps have been reported to be associated with calcium or cholesterol crystals and monosodium urate crystal-related diseases (45, 46), but are limited in CaOx crystal disease. Makki et al. showed an increase in neutrophil infiltration in the papillae of patients with brushite *versus* CaOx (47). Conversely, we identified that CD45^+^CD11b^+^GR1^+^ neutrophils, which aggravated crystal adhesion, were increased in the kidneys of CaOx mice. However, the mechanism of neutrophils in CaOx stone formation needs to be explored in further studies.

SCFAs are metabolites resulting from gut bacteria fermentation that are composed of fatty acids with fewer than six carbons (such as acetic acid, propionic acid, and butyric acid), which account for over 90% of all SCFAs (48, 49). SCFAs can regulate the functions of almost all immune cells by altering their differentiation, chemotaxis, and proliferation through SCFA receptors, including G protein-coupled receptors (48, 50). GPR43, also known as free fatty acid receptor 2 (FFAR2), has been shown to be involved in immune responses and inflammation management (39). Recent data revealed that butyrate facilitates M2 macrophage polarization *in vitro* and *in vivo* (51). Nakajima et al. reported that GPR43 activation by SCFAs leads to TNF-α induction in anti-inflammatory M2 macrophages located within adipose tissue (52). Vieira et al. observed that acetate restored the responsiveness of macrophages to MSU crystals in a GPR43-dependent manner (53). Consistent with previous studies, our results demonstrated that SCFAs increased the frequency of CX3CR1^+^CD24^-^ macrophages and suppressed crystal adhesion to TCMK-1 cells in a GPR43-dependent manner. However, Kamp et al. found that SCFA modulated neutrophil recruitment during inflammatory responses *via* GPR43, and more intravascular neutrophil rolling and adhesion was observed in GPR43-deficient mice in response to LPS (54). In this study, more GR1^+^ neutrophils infiltrated kidneys with CaOx crystals, but this process was inhibited by SCFAs; however, SCFAs could not modulate neutrophil infiltration in GPR43 inhibitor-treated mice. These results indicate that SCFAs have an inhibitory effect on neutrophils. Consistent with the results of other studies showing that SCFAs reduced CXCR2 expression in neutrophils and CXCL-2 production by neutrophils, our current findings suggest that SCFAs exerted an inhibitory effect on neutrophils (55, 56).

Neutrophils are recruited to sites of injury in response to danger-associated molecular patterns in order to phagocytose and produce cytokines, which leads to inflammation (57, 58). After neutrophils arrive at the site of injury, they can initiate neutrophil–macrophage crosstalk (59). Interestingly, we found neutrophil–macrophage crosstalk occurred *via* GPR43. CX3CR1^+^CD24^-^ macrophages not only regulated GPR43 expression in neutrophils, but also modulated neutrophil migration through GPR43.

In conclusion, 29 distinct immune cell types were identified in the kidneys of mice with CaOx crystals, in which CX3CR1^+^CD24^-^ macrophages significantly decreased and GR1^+^ neutrophils significantly increased. SCFAs inhibited CaOx crystal formation in the kidney by modulating the functions of CX3CR1^+^CD24^-^ macrophages and GR1^+^ neutrophils in a manner that depended on the microbiota and GPR43. These findings suggest that future immunotherapies may involve the use of SCFAs to prevent the formation of kidney stones.

## Data Availability Statement

The datasets presented in this study can be found in online repositories. The names of the repository/repositories and accession number(s) can be found in the article/**Supplementary Material**.

## Ethics Statement

The animal study was reviewed and approved by the Animal Research Ethics Committee of the West China Hospital in Sichuan University.

## Author Contributions

XJ and ZJ conceived and designed the study, analyzed the data, and wrote the first draft of the manuscript. XC, YM, HM, and YL conducted animal experiments and collected samples. LG and LX conducted laboratory experiments. LX, SZ, and XS analyzed the data. SQ, HL, and KW participated in manuscript revision. KW designed the study and made the final draft. All authors contributed to the article and approved the submitted version.

## Funding

This study was supported by the National Natural Science Foundation of China [81970602, 81770703], the Foundation of Science & Technology Department of Sichuan Province [2021YFS0116], the 1·3·5 Project for Disciplines of Excellence, West China Hospital, Sichuan University [ZYJC18015, ZYGD18011], and the Post-Doctor Research Project, West China Hospital, Sichuan University [2019HXBH087].

## Conflict of Interest

The authors declare that the research was conducted in the absence of any commercial or financial relationships that could be construed as a potential conflict of interest.

## Publisher’s Note

All claims expressed in this article are solely those of the authors and do not necessarily represent those of their affiliated organizations, or those of the publisher, the editors and the reviewers. Any product that may be evaluated in this article, or claim that may be made by its manufacturer, is not guaranteed or endorsed by the publisher.

## References

[1] Sayer JA. Progress in Understanding the Genetics of Calcium-Containing Nephrolithiasis. J Am Soc Nephrol (2017) 28(3):748–9. doi: 10.1681/ASN.2016050576 PMC5328168 27932479

[2] Khan A. Prevalence, Pathophysiological Mechanisms and Factors Affecting Urolithiasis. Int Urol Nephrol (2018) 50(5):799–806. doi: 10.1007/s11255-018-1849-2 29569213

[3] Liu Y Chen Y Liao B Luo D Wang K Li H. Epidemiology of Urolithiasis in Asia. Asian J Urol (2015) 5(4):205–14. doi: 10.1016/j.ajur.2018.08.007 PMC6197415 30364478

[4] Zisman AL. Effectiveness of Treatment Modalities on Kidney Stone Recurrence. Clin J Am Soc Nephrol (2017) 12(10):1699–708. doi: 10.2215/CJN.11201016 PMC5628726 28830863

[5] Howles SA Thakker RV. Genetics of Kidney Stone Disease. Nat Rev Urol (2020) 17(7):407–21. doi: 10.1038/s41585-020-0332-x 32533118

[6] Mulay SR Kulkarni OP Rupanagudi KV Migliorini A Darisipudi MN Vilaysane A. Calcium Oxalate Crystals Induce Renal Inflammation by NLRP3-Mediated IL-1beta Secretion. J Clin Invest (2013) 123(1):236246. doi: 10.1172/JCI63679 PMC3533282 23221343

[7] Mulay SR Desai J Kumar SV Thomasova D Romoli S Grigorescu M. Cytotoxicity of Crystals Involves RIPK3-MLKL-Mediated Necroptosis. Nat Commun (2016) 7:10274. doi: 10.1038/ncomms10274 26817517 PMC4738349

[8] Duewell P Kono H Rayner KJ Sirois CM Vladimer G Bauernfeind FG. NLRP3 Inflammasomes Are Required for Atherogenesis and Activated by Cholesterol Crystals. Nature (2010) 464(7923):1357–61. doi: 10.1038/nature08938 PMC2946640 20428172

[9] Sun Y Liu Y Guan X Kang J Wang X Liu Q. Atorvastatin Inhibits Renal Inflammatory Response Induced by Calcium Oxalate Crystals via Inhibiting the Activation of TLR4/NF-kappaB and NLRP3 Inflammasome. IUBMB Life (2020) 72(5):1065–74. doi: 10.1002/iub.2250 32083808

[10] Joshi S Wang W Peck AB Khan SR. Activation of the NLRP3 Inflammasome in Association With Calcium Oxalate Crystal Induced Reactive Oxygen Species in Kidneys. J Urol (2015) 193(5):1684–91. doi: 10.1016/j.juro.2014.11.093 PMC4406847 25437532

[11] Okada A Yasui T Hamamoto S Hirose M Kubota Y Itoh Y. Genome-Wide Analysis of Genes Related to Kidney Stone Formation and Elimination in the Calcium Oxalate Nephrolithiasis Model Mouse: Detection of Stone-Preventive Factors and Involvement of Macrophage Activity. J Bone Miner Res (2009) 24(5):908–24. doi: 10.1359/jbmr.081245 19113933

[12] Taguchi K Hamamoto S Okada A Unno R Kamisawa H Naiki T. Genome-Wide Gene Expression Profiling of Randall’s Plaques in Calcium Oxalate Stone Formers. J Am Soc Nephrol (2017) 28(1):333–47. doi: 10.1681/ASN.2015111271 PMC5198277 27297950

[13] Anders HJ Suarez-Alvarez B Grigorescu M Foresto-Neto O Steiger S Desai J. The Macrophage Phenotype and Inflammasome Component NLRP3 Contributes to Nephrocalcinosis-Related Chronic Kidney Disease Independent From IL-1-Mediated Tissue Injury. Kidney Int (2018) 93(3):656–69. doi: 10.1016/j.kint.2017.09.022 29241624

[14] Xi J Chen Y Jing J Zhang Y Liang C Hao Z. Sirtuin 3 Suppresses the Formation of Renal Calcium Oxalate Crystals Through Promoting M2 Polarization of Macrophages. J Cell Physiol (2019) 234(7):11463–73. doi: 10.1002/jcp.27803 30588609

[15] Luz HL Reichel M Unwin RJ Mutig K Najenson AC Tonner LM. P2X7 Receptor Stimulation Is Not Required for Oxalate Crystal-Induced Kidney Injury. Sci Rep (2019) 9(1):20086. doi: 10.1038/s41598-019-56560-2 31882798 PMC6934555

[16] Zhu C Liang Q Liu Y Kong D Zhang J Wang H. Kidney Injury in Response to Crystallization of Calcium Oxalate Leads to Rearrangement of the Intrarenal T Cell Receptor Delta Immune Repertoire. J Transl Med (2019) 17(1):278. doi: 10.1186/s12967-019-2022-0 31438987 PMC6704580

[17] Kusmartsev S Dominguez-Gutierrez PR Canales BK Bird VG Vieweg J Khan SR. Calcium Oxalate Stone Fragment and Crystal Phagocytosis by Human Macrophages. J Urol (2016) 195(4 Pt 1):1143–51. doi: 10.1016/j.juro.2015.11.048 PMC4882284 26626217

[18] Liu Y Jin X Hong HG Xiang L Jiang Q Ma Y. The Relationship Between Gut Microbiota and Short Chain Fatty Acids in the Renal Calcium Oxalate Stones Disease. FASEB J (2020) 34(8):11200–14. doi: 10.1096/fj.202000786R 32645241

[19] Flossmann V Cross A Issa F. Propionic Acid Shapes the Multiple Sclerosis Disease Course by an Immunomodulatory Mechanism. Transplantation (2020) 180(6):1067–80. doi: 10.1016/j.cell.2020.02.035 32160527

[20] Lavelle A Sokol H. Gut Microbiota-Derived Metabolites as Key Actors in Inflammatory Bowel Disease. Nat Rev Gastro Hepat (2020) 17(4):223–37. doi: 10.1038/s41575-019-0258-z 32076145

[21] Fachi JL Sécca C Rodrigues PB Mato FCP Di Luccia B Felipe JS. Acetate Coordinates Neutrophil and ILC3 Responses Against C. Difficile Through FFAR2. J Exp Med (2020) 217(3):jem.20190489. doi: 10.1084/jem.20190489 PMC7062529 31876919

[22] Furusawa Y Obata Y Fukuda S Endo TA Nakato G Takahashi D. Commensal Microbe-Derived Butyrate Induces the Differentiation of Colonic Regulatory T Cells. Nature (2013) 504(7480):446–50. doi: 10.1038/nature12721 24226770

[23] Arpaia N Campbell C Fan X Dikiy S van der Veeken J deRoos P. Metabolites Produced by Commensal Bacteria Promote Peripheral Regulatory T-Cell Generation. Nature (2013) 504(7480):451–55. doi: 10.1038/nature12726 PMC3869884 24226773

[24] Ohno H. The Impact of Metabolites Derived From the Gut Microbiota on Immune Regulation and Diseases. Int Immunol (2020) 32(10):629–36. doi: 10.1093/intimm/dxaa041 32564086

[25] Taguchi K Chen L Usawachintachit M Hamamoto S Kang M Sugino T. Fatty Acid-Binding Protein 4 Downregulation Drives Calcification in the Development of Kidney Stone Disease. Kidney Int (2020) 97(5):1042–56. doi: 10.1016/j.kint.2020.01.042 32247632

[26] Liu H Yang X Tang K Ye T Duan C Lv P. Sulforaphane Elicts Dual Therapeutic Effects on Renal Inflammatory Injury and Crystal Deposition in Calcium Oxalate Nephrocalcinosis. Theranostics (2020) 10(16):7319–34. doi: 10.7150/thno.44054 PMC7330860 32641994

[27] Cao Q Wang Y Niu Z Wang C Wang R Zhang Z. Potentiating Tissue-Resident Type 2 Innate Lymphoid Cells by IL-33 to Prevent Renal Ischemia-Reperfusion Injury. J Am Soc Nephrol (2018) 29(3):961–76. doi: 10.1681/ASN.2017070774 PMC5827602 29295873

[28] Samusik N Good Z Spitzer MH Davis KL Nolan GP. Automated Mapping of Phenotype Space With Single-Cell Data. Nat Methods (2016) 13(6):493–6. doi: 10.1038/nmeth.3863 PMC4896314 27183440

[29] van der Maaten L Hinton G. Visualizing Data Using T-SNE. J Mach Learn Res (2008) 9:2579–605.

[30] Trapnell C Pachter L Salzberg SL. TopHat: Discovering Splice Junctions With RNA-Seq. Bioinformatics (2009) 25(9):1105–11. doi: 10.1093/bioinformatics/btp120 PMC2672628 19289445

[31] Li B Dewey CN. RSEM: Accurate Transcript Quantification From RNA-Seq Data With or Without a Reference Genome. BMC Bioinf (2011) 12:323. doi: 10.1186/1471-2105-12-323 PMC3163565 21816040

[32] Robinson MD McCarthy DJ Smyth GK. Edger: A Bioconductor Package for Differential Expression Analysis of Digital Gene Expression Data. Bioinformatics (2010) 26(1):139–40. doi: 10.1093/bioinformatics/btp616 PMC2796818 19910308

[33] Chen SF Zhou YQ Chen YR Gu J. Fastp: An Ultra-Fast All-in-One FASTQ Preprocessor. Bioinformatics (2018) 34(17):884–90. doi: 10.1093/bioinformatics/bty560 PMC6129281 30423086

[34] Magoc T Salzberg SL. FLASH: Fast Length Adjustment of Short Reads to Improve Genome Assemblies. Bioinformatics (2011) 27(21):2957–63. doi: 10.1093/bioinformatics/btr507 PMC3198573 21903629

[35] Edgar RC. UPARSE: Highly Accurate OTU Sequences From Microbial Amplicon Reads. Nat Methods (2013) 10(10):996–8. doi: 10.1038/nmeth.2604 23955772

[36] Wang Q Garrity GM Tiedje JM Cole JR. Naive Bayesian Classifier for Rapid Assignment of rRNA Sequences Into the New Bacterial Taxonomy. Appl Environ Microb (2007) 73(16):5261–7. doi: 10.1128/AEM.00062-07 PMC1950982 17586664

[37] De Filippo K Dudeck A Hasenberg M Nye E van Rooijen N Hartmann K. Mast Cell and Macrophage Chemokines CXCL1/CXCL2 Control the Early Stage of Neutrophil Recruitment During Tissue Inflammation. Blood (2013) 121(24):4930–7. doi: 10.1182/blood-2013-02-486217 23645836

[38] McLoughlin RM Witowski J Robson RL Wilkinson TS Hurst SM Williams AS. Interplay Between IFN-Gamma and IL-6 Signaling Governs Neutrophil Trafficking and Apoptosis During Acute Inflammation. J Clin Invest (2003) 112(4):598–607. doi: 10.1172/JCI17129 12925700 PMC171385

[39] Brown AJ Goldsworthy SM Barnes AA Eilert MM Tcheang L Daniels D. The Orphan G Protein-Coupled Receptors GPR41 and GPR43 Are Activated by Propionate and Other Short Chain Carboxylic Acids. J Biol Chem (2003) 278(13):11312–9. doi: 10.1074/jbc.M211609200 12496283

[40] Nilsson NE Kotarsky K Owman C Olde B. Identification of a Free Fatty Acid Receptor, FFA2R, Expressed on Leukocytes and Activated by Short-Chain Fatty Acids. Biochem Biophys Res Commun (2003) 303(4):1047–52. doi: 10.1016/s0006-291x(03)00488-1 12684041

[41] Kim CH. Immune Regulation by Microbiome Metabolites. Immunology (2018) 154(2):220–9. doi: 10.1111/imm.12930 PMC5980225 29569377

[42] Dominguez-Gutierrez PR Kwenda EP Khan SR Canales BK. Immunotherapy for Stone Disease. Curr Opin Urol (2020) 30(2):183–9. doi: 10.1097/MOU.0000000000000729 31913203

[43] Zhao W Lu H Wang X Ransohoff RM Zhou L. CX3CR1 Deficiency Delays Acute Skeletal Muscle Injury Repair by Impairing Macrophage Functions. FASEB J (2016) 30(1):380–93. doi: 10.1096/fj.14-270090 PMC4684523 26443824

[44] Marelli G Belgiovine C Mantovani A Erreni M Allavena P. Non-Redundant Role of the Chemokine Receptor CX3CR1 in the Anti-Inflammatory Function of Gut Macrophages. Immunobiology (2017) 222(2):463–72. doi: 10.1016/j.imbio.2016.07.013 27707514

[45] Munoz LE Boeltz S Bilyy R Schauer C Mahajan A Widulin N. Neutrophil Extracellular Traps Initiate Gallstone Formation. Immunity (2019) 51(3):443–50.e4. doi: 10.1016/j.immuni.2019.07.002 31422870

[46] Schauer C Janko C Munoz LE Zhao Y Kienhöfer D Frey B. Aggregated Neutrophil Extracellular Traps Limit Inflammation by Degrading Cytokines and Chemokines. Nat Med (2014) 20(5):511–7. doi: 10.1038/nm.3547 24784231

[47] Makki MS Winfree S Lingeman JE Witzmann FA Worcester EM Krambeck AE. A Precision Medicine Approach Uncovers a Unique Signature of Neutrophils in Patients With Brushite Kidney Stones. Kidney Int Rep (2020) 5(5):663–7. doi: 10.1016/j.ekir.2020.02.1025 PMC7210605 32405588

[48] Sun MM Wu W Liu ZJ Cong Y. Microbiota Metabolite Short Chain Fatty Acids, GPCR, and Inflammatory Bowel Diseases. J Gastroenterol (2017) 52(1):1–8. doi: 10.1007/s00535-016-1242-9 27448578 PMC5215992

[49] Li L Ma L Fu P. Gut Microbiota-Derived Short-Chain Fatty Acids and Kidney Diseases. Drug Des Devel Ther (2017) 11:3531–42. doi: 10.2147/DDDT.S150825 PMC5729884 29270002

[50] Kimura I Ichimura A Ohue-Kitano R Igarashi M. Free Fatty Acid Receptors in Health and Disease. Physiol Rev (2020) 100(1):171–210. doi: 10.1152/physrev.00041.2018 31487233

[51] Ji J Shu D Zheng M Wang J Luo C Wang Y. Microbial Metabolite Butyrate Facilitates M2 Macrophage Polarization and Function. Sci Rep (2016) 6:24838. doi: 10.1038/srep24838 27094081 PMC4837405

[52] Nakajima A Nakatani A Hasegawa S Irie J Ozawa K Tsujimoto G. The Short Chain Fatty Acid Receptor GPR43 Regulates Inflammatory Signals in Adipose Tissue M2-Type Macrophages. PloS One (2017) 12(7):e0179696. doi: 10.1371/journal.pone.0179696 28692672 PMC5503175

[53] Vieira AT Macia L Galvao I Martins FS Canesso MC Amaral FA. A Role for Gut Microbiota and the Metabolite-Sensing Receptor GPR43 in a Murine Model of Gout. Arthritis Rheumatol (2015) 67(6):1646–56. doi: 10.1002/art.39107 25914377

[54] Kamp ME Shim R Nicholls AJ Oliveira AC Mason LJ Binge L. G Protein-Coupled Receptor 43 Modulates Neutrophil Recruitment During Acute Inflammation. PloS One (2016) 11(9):e0163750. doi: 10.1371/journal.pone.0163750 27658303 PMC5033414

[55] Maslowski KM Vieira AT Ng A Kranich J Sierro F Yu D. Regulation of Inflammatory Responses by Gut Microbiota and Chemoattractant Receptor GPR43. Nature (2009) 461(7268):1282–6. doi: 10.1038/nature08530 PMC3256734 19865172

[56] Vinolo MAR Rodrigues HG Hatanaka E Sato FT Sampaio SC. Curi R Suppressive Effect of Short-Chain Fatty Acids on Production of Proinflammatory Mediators by Neutrophils. J Nutr Biochem (2011) 22(9):849–55. doi: 10.1016/j.jnutbio.2010.07.009 21167700

[57] de Oliveira S Rosowski EE Huttenlocher A. Neutrophil Migration in Infection and Wound Repair: Going Forward in Reverse. Nat Rev Immunol (2016) 16(6):378–91. doi: 10.1038/nri.2016.49 PMC5367630 27231052

[58] Kolaczkowska E Kubes P. Neutrophil Recruitment and Function in Health and Inflammation. Nat Rev Immunol (2013) 13(3):159–75. doi: 10.1038/nri3399 23435331

[59] Bouchery T Harris N. Neutrophil-Macrophage Cooperation and Its Impact on Tissue Repair. Immunol Cell Bio (2019) l97(3):289–98. doi: 10.1111/imcb.12241 30710448

