# Supplementary material for: Short Chain Fatty Acids Prevent Glyoxylate-Induced Calcium Oxalate Stones by GPR43-Dependent Immunomodulatory Mechanism
Source: Front Immunol. 2021 Oct 5;12:729382. doi: 10.3389/fimmu.2021.729382 (PMC8523925; doi:10.3389/fimmu.2021.729382)
Supplement: Supplementary file 1 [file DataSheet_1.docx]

**Supplementary Material**

**Supplementary Tables**

Supplemental Table 1 Markers of mass cytometry

| Number | Marker | Number | Marker |
| --- | --- | --- | --- |
| 1 | CD45 | 22 | Ly6G |
| 2 | CD3 | 23 | CD24 |
| 3 | CD4 | 24 | ST2 |
| 4 | CD8 | 25 | MHC II |
| 5 | CD19 | 26 | CD172 |
| 6 | gd TCR | 27 | B220 |
| 7 | CD62L | 28 | BST2 |
| 8 | CD25 | 29 | CD103 |
| 9 | FoxP3 | 30 | CD90 |
| 10 | F4/80 | 31 | CD206 |
| 11 | CD11b | 32 | PD-1 |
| 12 | CD68 | 33 | CD56 |
| 13 | CD14 | 34 | ICOS |
| 14 | INOS | 35 | CXCR3 |
| 15 | Arg1 | 36 | gata-3 |
| 16 | Ly6C | 37 | CD44 |
| 17 | CD127 | 38 | NKp46 |
| 18 | CX3CR1 | 39 | CD11c |
| 19 | CCR2 | 40 | NK1.1 |
| 20 | MERTK | 41 | CD69 |
| 21 | Ly6G/C(Gr1) | 42 | CD49a |

Supplemental Table 2 Immune-cell populations in different clusters

| **Subsets Label** |  | **Cluster** |
| --- | --- | --- |
| T cells | CD11c+ CD8 T cells | 1 |
|  | Ly6C+ CD8 T cells | 2 |
|  | Effector CD8 T cells | 3 |
|  | naive CD8 T cells | 4 |
|  | CCR2+ CD4 T cells | 5 |
|  | Activated CD4 T cells | 6 |
|  | CD69- CD4 T cells | 7 |
|  | Effector CD4 T cells | 8 |
|  | Double negative T cells | 9 |
|  | γδ T cells | 11 |
| B cells | BST2+ B cells | 13 |
|  | BST2- B cells | 16 |
| Macrophages | SIRPα+ macrophages | 21 |
|  | **CD11c+ CX3CR1+ macrophages** | **22** |
|  | **CD11c- CX3CR1+ macrophages** | **23** |
|  | M2 | 24 |
|  | CD25+ macrophages | 25 |
| Monocytes | Ly6C+ monocytes | 26 |
| DCs | CD103+ DCs | 17 |
|  | CD103- DCs | 18 |
|  | CD11c+CD11b+ DCs | 27 |
|  | Plasmacytoid DCs(BTS2+) | 28 |
|  | CD14^+^ DCs | 29 |
|  | cDCs | 14 |
| NK | NK cells | 19 |
| Neutrophil | **Neutrophils** | **20** |
| ILCs | ILC2 | 10 |
| Unknown cells | Ly6C+ BST2+ Cells | 12 |
|  | CD69+ CD49a+ CCR2+ Cells | 15 |

Supplemental Table 3 Higher bacteria genera in controls, CaOx, C2, C3 and C4 groups by LEfSe analysis.

| Species name | group | Mean | LDA_value | Pvalue |
| --- | --- | --- | --- | --- |
| p__Proteobacteria.c__Alphaproteobacteria.o__Caulobacterales.f__Caulobacteraceae.g__Brevundimonas | C2 | 2.537 | 3.645 | 0.025 |
| p__Firmicutes.c__Clostridia.o__Clostridia_vadinBB60_group.f__norank_o__Clostridia_vadinBB60_group.g__norank_f__norank_o__Clostridia_vadinBB60_group | C2 | 3.212 | 3.151 | 0.040 |
| p__Proteobacteria.c__Gammaproteobacteria.o__Enterobacterales.f__Enterobacteriaceae.g__Enterobacter | C2 | 3.664 | 3.352 | 0.049 |
| p__Firmicutes.c__Clostridia.o__Clostridiales.f__Clostridiaceae.g__Candidatus_Arthromitus | C2 | 3.084 | 3.362 | 0.017 |
| p__Firmicutes.c__Clostridia.o__Clostridia_vadinBB60_group.f__norank_o__Clostridia_vadinBB60_group | C2 | 3.212 | 3.151 | 0.040 |
| p__Firmicutes.c__Clostridia.o__Clostridia_vadinBB60_group | C2 | 3.212 | 3.151 | 0.040 |
| p__Patescibacteria.c__Saccharimonadia.o__Saccharimonadales.f__Saccharimonadaceae | C3 | 4.435 | 4.114 | 0.013 |
| p__Patescibacteria.c__Saccharimonadia.o__Saccharimonadales.f__Saccharimonadaceae.g__Candidatus_Saccharimonas | C3 | 4.435 | 4.114 | 0.013 |
| p__Actinobacteriota.c__Actinobacteria.o__Bifidobacteriales.f__Bifidobacteriaceae | C3 | 4.482 | 4.073 | 0.032 |
| p__Actinobacteriota.c__Actinobacteria.o__Propionibacteriales.f__Propionibacteriaceae.g__Cutibacterium | C3 | 1.999 | 4.458 | 0.022 |
| p__Firmicutes.c__Bacilli.o__Erysipelotrichales.f__Erysipelotrichaceae.g__Turicibacter | C3 | 3.777 | 3.609 | 0.007 |
| p__Actinobacteriota | C3 | 4.798 | 4.311 | 0.007 |
| p__Actinobacteriota.c__Actinobacteria.o__Bifidobacteriales | C3 | 4.482 | 4.073 | 0.032 |
| p__Actinobacteriota.c__Actinobacteria.o__Bifidobacteriales.f__Bifidobacteriaceae.g__Bifidobacterium | C3 | 4.482 | 4.073 | 0.032 |
| p__Patescibacteria | C3 | 4.439 | 4.116 | 0.015 |
| p__Patescibacteria.c__Saccharimonadia | C3 | 4.437 | 4.115 | 0.013 |
| p__Patescibacteria.c__Saccharimonadia.o__Saccharimonadales | C3 | 4.437 | 4.115 | 0.013 |
| p__Firmicutes.c__Bacilli.o__Erysipelotrichales.f__Erysipelotrichaceae.g__Faecalibaculum | C4 | 4.589 | 4.229 | 0.042 |
| p__Firmicutes.c__Clostridia.o__Oscillospirales.f__Ruminococcaceae.g__Ruminococcus | C4 | 3.108 | 3.285 | 0.031 |
| p__Bacteroidota.c__Bacteroidia.o__Bacteroidales.f__Prevotellaceae.g__unclassified_f__Prevotellaceae | C4 | 3.555 | 3.452 | 0.001 |
| p__Actinobacteriota.c__Coriobacteriia.o__Coriobacteriales | Control | 4.637 | 4.140 | 0.036 |
| p__Actinobacteriota.c__Coriobacteriia.o__Coriobacteriales.f__Eggerthellaceae.g__unclassified_f__Eggerthellaceae | Control | 3.886 | 3.523 | 0.012 |
| p__Actinobacteriota.c__Coriobacteriia | Control | 4.637 | 4.140 | 0.036 |
| p__Actinobacteriota.c__Coriobacteriia.o__Coriobacteriales.f__Eggerthellaceae | Control | 4.612 | 4.125 | 0.034 |
| p__Firmicutes.c__Clostridia.o__Lachnospirales.f__Lachnospiraceae.g__ASF356 | Control | 2.328 | 4.014 | 0.013 |
| p__Firmicutes.c__Bacilli.o__Erysipelotrichales.f__Erysipelotrichaceae.g__Dubosiella | Control | 5.087 | 4.708 | 0.008 |
| p__Firmicutes.c__Clostridia.o__unclassified_c__Clostridia.f__unclassified_c__Clostridia.g__unclassified_c__Clostridia | CaOx | 2.300 | 3.768 | 0.003 |
| p__Bacteroidota.c__Bacteroidia.o__Bacteroidales.f__Rikenellaceae.g__Alistipes | CaOx | 4.143 | 3.745 | 0.037 |
| p__Bacteroidota.c__Bacteroidia.o__Bacteroidales.f__Rikenellaceae | CaOx | 4.296 | 3.895 | 0.015 |
| p__Firmicutes.c__Clostridia.o__Peptococcales.f__Peptococcaceae | CaOx | 2.942 | 3.360 | 0.006 |
| p__Firmicutes.c__Clostridia.o__unclassified_c__Clostridia | CaOx | 2.300 | 3.788 | 0.003 |
| p__Bacteroidota.c__Bacteroidia.o__Bacteroidales.f__Rikenellaceae.g__Rikenellaceae_RC9_gut_group | CaOx | 3.717 | 3.422 | 0.003 |
| p__Actinobacteriota.c__Coriobacteriia.o__Coriobacteriales.f__Atopobiaceae.g__Coriobacteriaceae_UCG_002 | CaOx | 3.511 | 3.180 | 0.038 |
| p__Bacteroidota.c__Bacteroidia.o__Bacteroidales.f__Rs_E47_termite_group.g__norank_f__Rs_E47_termite_group | CaOx | 3.292 | 3.286 | 0.010 |
| p__Bacteroidota.c__Bacteroidia.o__Bacteroidales.f__Rs_E47_termite_group | CaOx | 3.292 | 3.286 | 0.010 |
| p__Firmicutes.c__Clostridia.o__Lachnospirales.f__Lachnospiraceae.g__Eubacterium_fissicatena_group | CaOx | 3.823 | 3.500 | 0.010 |
| p__Firmicutes.c__Bacilli.o__Erysipelotrichales.f__Erysipelotrichaceae | CaOx | 5.248 | 4.787 | 0.027 |
| p__Firmicutes.c__Bacilli.o__Erysipelotrichales.f__Erysipelotrichaceae.g__Allobaculum | CaOx | 5.134 | 4.802 | 0.002 |
| p__Firmicutes.c__Clostridia.o__Peptococcales.f__Peptococcaceae.g__norank_f__Peptococcaceae | CaOx | 2.942 | 3.355 | 0.006 |
| p__Firmicutes.c__Clostridia.o__unclassified_c__Clostridia.f__unclassified_c__Clostridia | CaOx | 2.300 | 3.832 | 0.003 |
| p__Firmicutes.c__Clostridia.o__Peptococcales | CaOx | 2.942 | 3.360 | 0.006 |
| p__Bacteroidota.c__Bacteroidia.o__Bacteroidales.f__Rikenellaceae.g__Rikenella | CaOx | 2.743 | 3.444 | 0.025 |
| p__Actinobacteriota.c__Coriobacteriia.o__Coriobacteriales.f__Atopobiaceae | CaOx | 3.517 | 3.187 | 0.037 |
| p__Firmicutes.c__Bacilli.o__Erysipelotrichales | CaOx | 5.255 | 4.790 | 0.034 |

Supplemental Table 4 Differentially abundant metabolic pathways among groups.

| Pathway.level3 | Description | Control | CaOx | C2 | C3 | C4 | p.value |
| --- | --- | --- | --- | --- | --- | --- | --- |
| ko02010 | ABC transporters | 1011404.095 | 1064278.814 | 1874705.045 | 1195238.774 | 1122782.845 | 0.035 |
| ko00190 | Oxidative phosphorylation | 306009.833 | 375337.3 | 486322.47 | 352111.913 | 344949.531 | 0.014 |
| ko00051 | Fructose and mannose metabolism | 307667.895 | 341552.86 | 486760.01 | 320902.415 | 316951.008 | 0.029 |
| ko02060 | Phosphotransferase system (PTS) | 297770.385 | 277498.36 | 408273.553 | 247651.246 | 205033.1 | 0.024 |
| ko00564 | Glycerophospholipid metabolism | 195634.648 | 215485.343 | 285601.366 | 204956.341 | 197642.823 | 0.017 |
| ko00061 | Fatty acid biosynthesis | 178954.758 | 207962.489 | 285727.58 | 202832.881 | 205918.421 | 0.048 |
| ko00340 | Histidine metabolism | 107342.255 | 149832.906 | 196701.74 | 136951.473 | 147417.659 | 0.032 |
| ko01503 | Cationic antimicrobial peptide (CAMP) resistance | 99071.073 | 107472.259 | 158499.911 | 103350.921 | 117535.779 | 0.019 |
| ko04122 | Sulfur relay system | 80610.835 | 93671.863 | 138018.423 | 88583.111 | 85834.756 | 0.007 |
| ko00280 | Valine, leucine and isoleucine degradation | 67868.771 | 83326.976 | 136454.394 | 81669.706 | 82016.218 | 0.017 |
| ko00071 | Fatty acid degradation | 64561.068 | 70528.67 | 109142.453 | 64698.458 | 65143.196 | 0.006 |
| ko00940 | Phenylpropanoid biosynthesis | 29615.304 | 48877.319 | 63385.658 | 42058.016 | 48592.133 | 0.041 |
| ko04727 | GABAergic synapse | 41108.501 | 43770.8 | 56089.731 | 45237.094 | 44351.265 | 0.025 |
| ko00401 | Novobiocin biosynthesis | 30875.518 | 41990.439 | 52536.071 | 38935.538 | 45020.664 | 0.032 |
| ko00380 | Tryptophan metabolism | 21686.831 | 27615.256 | 64684.998 | 26551.481 | 27181.103 | 0.014 |
| ko00960 | Tropane, piperidine and pyridine alkaloid biosynthesis | 23032.884 | 31201.269 | 40058.565 | 28559.221 | 35453.795 | 0.01 |
| ko04213 | Longevity regulating pathway - multiple species | 21431.139 | 25679.236 | 42144.085 | 26353.399 | 30377.784 | 0.026 |
| ko00785 | Lipoic acid metabolism | 11835.665 | 13443.61 | 29168.01 | 16331.351 | 16250.966 | 0.022 |
| ko00903 | Limonene and pinene degradation | 6264.019 | 10087.186 | 17504.311 | 8318.574 | 10367.278 | 0.046 |
| ko04068 | FoxO signaling pathway | 6036.138 | 8171.525 | 17757.694 | 9488.103 | 10058.083 | 0.034 |
| ko04211 | Longevity regulating pathway | 4773.911 | 6777.373 | 16999.453 | 7900.873 | 8747.998 | 0.014 |
| ko00361 | Chlorocyclohexane and chlorobenzene degradation | 6414.085 | 5800.953 | 11752.618 | 6067.399 | 6064.741 | 0.037 |
| ko00281 | Geraniol degradation | 2871.798 | 5847.539 | 9689.985 | 4948.058 | 8760.885 | 0.009 |
| ko00592 | alpha-Linolenic acid metabolism | 2006.995 | 3004.524 | 5872.248 | 2812.598 | 4292.461 | 0.04 |

**Supplementary Figures**

**
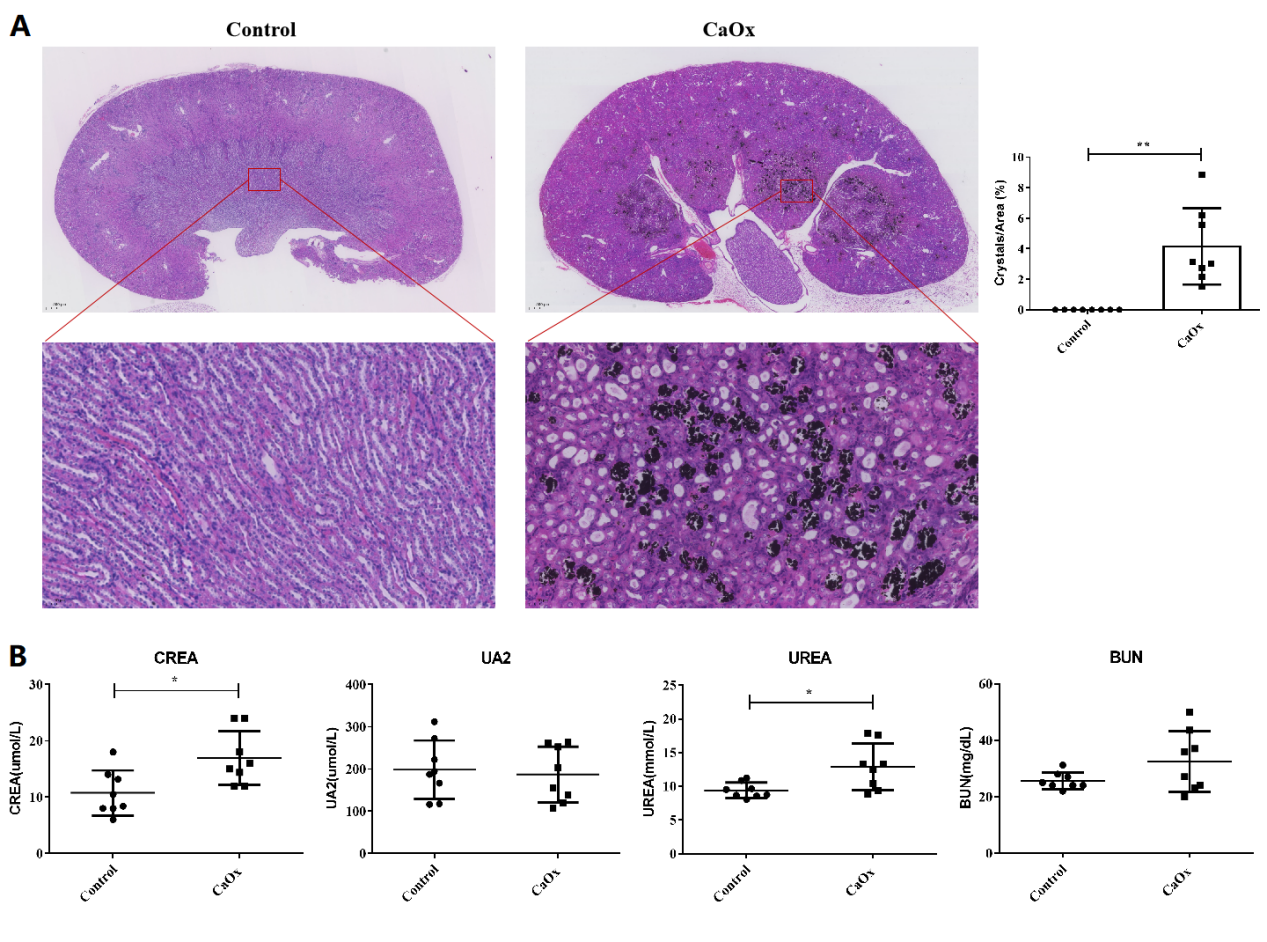
**

**Supplementary Figure 1 Renal calcium oxalate crystals established by glyoxylate.** The level of calcium oxalate crystals was detected by Von Kossa staining and Hematoxylin & Eosin (HE) staing. (**A**) Representative images of Von Kossa staining and HE staining in kidney. (**B**) Serum creatinine, uric acid, urea and blood urea nitrogen in mice were detected. Data are expressed as mean ± sd, *P < 0.05 and **P <0 .01.


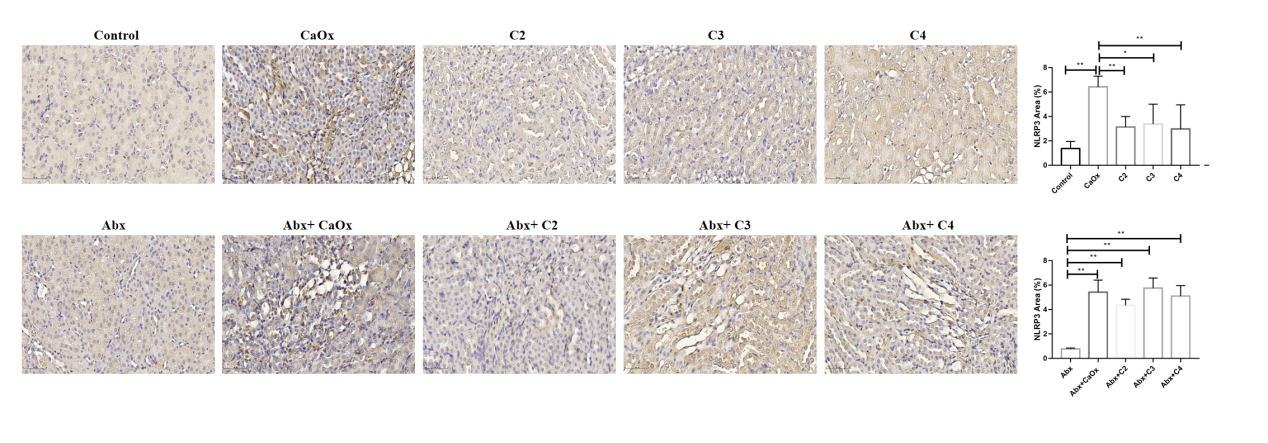


**Supplementary Figure 2 NLRP3 expression in kidney.** NLRP3 expression in kidney tissue was tested by IHC. Scale 50 μm. *p <0.05 and **p < 0.01.


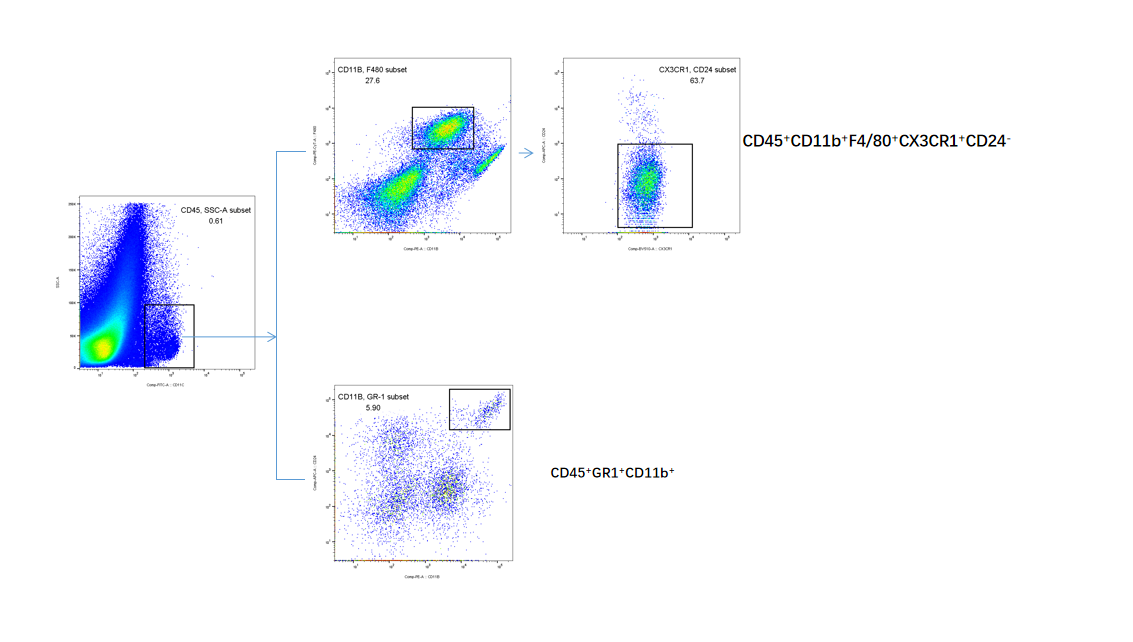


**Supplementary Figure 3** **Gating strategy to identify CD45^+^F4/80^+^CD11b^+^CX3CR1^+^CD24^-^ macrophages and CD45^+^CD11b^+^GR1^+^ neutrophils.** Cell were firstly gated on CD45^+^ cells, and neutrophills were gated on CD11b^+^GR1^+^ cells. Macrophages were then gated on F4/80^+^CD11b^+^ cells, and then gated on CX3CR1^+^CD24^-^ cells.


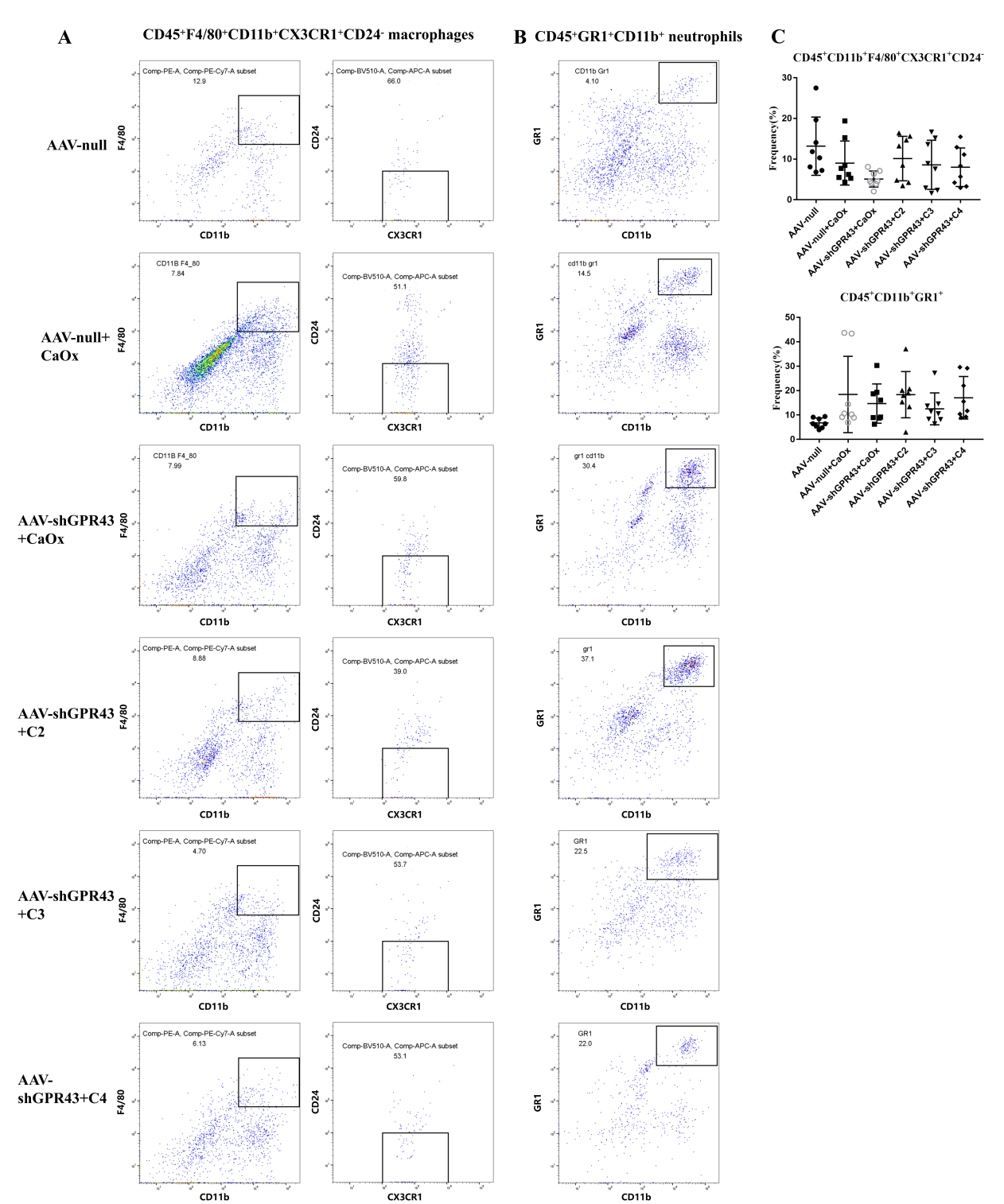


**Supplementary Figure 4** **Representative flow cytometric analysis of CD45^+^F4/80^+^CD11b^+^CX3CR1^+^CD24^-^ macrophages and CD45^+^GR1^+^CD11b^+^ neutrophil in CaOx crystal mice with administration of SCFAs after treatment of AAV carrying shRNA targeting GPR43.** AAV-shGPR43 represent as groups of mice treated with AAV carrying shRNA targeting GPR43 and administration with SCFAs. (**A**) macrophages were first gated on CD45^+^ cells, and then were gated on F4/80^+^CD11b^+^ cells followed by CX3CR1^+^CD24^-^, and (**B**) neutrophil were gated as GR1^+^CD11b^+^ in CD45^+^cells. (**C**) Frequencies of CD45^+^F4/80^+^CD11b^+^CX3CR1^+^CD24^-^ macrophages and CD45^+^GR1^+^CD11b^+^ neutrophil in kidney with administration of SCFAs. Data were shown as mean ± standard deviation. Data were analyzed by one-way ANOVA followed by Tukey’s multiple comparisons test among multiple groups.
